# Oncolytic peptide LTX-315 plus an anti-CTLA-4 antibody induces a synergistic anti-cancer immune response in residual tumors after radiofrequency ablation of hepatocellular carcinoma

**DOI:** 10.1038/s41419-025-07622-z

**Published:** 2025-04-13

**Authors:** Bo Sun, Jiayun Liu, Xiaocui Liu, Jing Li, Guilin Zhang, Tao Sun, Chuansheng Zheng, Xuefeng Kan

**Affiliations:** 1 Department of Radiology, Union Hospital, Tongji Medical College, Huazhong University of Science and Technology, Wuhan, 430022 China; 2 Hubei Provincial Clinical Research Center for Precision Radiology & Interventional Medicine, Wuhan, China; 3 Hubei Province Key Laboratory of Molecular Imaging, Wuhan, China

**Keywords:** Drug development, Cancer immunotherapy

## Abstract

Preventing tumor recurrence after radiofrequency ablation (RFA) of malignant solid tumors with large size or in high-risk locations represents a great challenge. In this study, we explored the feasibility of using oncolytic peptide LTX-315 plus an anti-cytotoxic T lymphocyte antigen-4 (CTLA-4) antibody for inhibiting residual tumors after RFA of hepatocellular carcinoma (HCC). In in vitro experiment, the CD8^+^T cells from Hepa1-6 tumors, after being subjected to three different treatments (control, iRFA, iRFA + LTX-315), were extracted and were then co-cultured with Hepa1-6 cells and an anti-CTLA-4 antibody. The enzyme-linked immunospot, flow cytometry, and cell counting kit-8 assay were employed to assess the cytotoxicity of extracted CD8^+^T cells on Hepa1-6 cells. In in vivo experiment, different murine orthotopic HCC models were variously treated by: (1) pseudo iRFA + phosphate-buffered saline (PBS); (2) iRFA + PBS; (3) iRFA + LTX-315; (4) iRFA + anti-CTLA-4 antibody; and (5) iRFA + LTX-315 + anti-CTLA-4 antibody. The treatment effects were compared among different groups and were pathologically confirmed. The possible mechanisms of the combination treatment (LTX-315+anti-CTLA-4 antibody) for residual tumors after iRFA of HCC were explored. LTX-315 significantly reduced the PD-1 expression and significantly increased CTLA-4 expression of CD8^+^T cells in residual tumors, and additional treatment of anti-CTLA-4 antibody could significantly enhance the cytotoxicity of CD8^+^T cells for Hepa1-6 cells in vitro experiments. Compared with the other treatments, the combined treatment of LTX-315 with anti-CTLA-4 antibody achieved a better tumor response and longer survival, and it could synergistically activate the cGAS-STING pathway and elicit an immunogenic cell death, leading to a strong anti-tumor immunity after iRFA of HCC. The immunosuppressive microenvironment of residual tumors was significantly improved by the combination therapy with a significantly increased ratio of M1-like tumor-associated macrophages to M2-like tumor-associated macrophages, a significantly decreased infiltration of regulatory T cells and myeloid-derived suppressor cells, and a significantly lower expression of PD-1 and CTLA-4. Overall, the results of this study demonstrated that LTX-315 plus anti-CTLA-4 antibody could synergistically improve the immunosuppressive microenvironment of residual tumors and induce a strong anti-tumor immunity after iRFA of HCC. This combination treatment strategy may offer a new alternative to reduce the tumor recurrence after RFA of malignant solid tumors with large sizes or in high-risk locations.

## Introduction

Radiofrequency ablation (RFA) is extensively applied for the treatment of early-stage hepatocellular carcinoma (HCC) [1], non-small cell lung cancer [2], and renal cell carcinomas [3], thanks to its minimal invasiveness and notable efficacy. Nevertheless, the 5-year tumor recurrence rate of these malignant solid tumors receiving RFA was not satisfactory [3, 4], which was mainly ascribed to the difficulty in achieving a complete ablation of the lesions. This is especially true of tumors with a diameter of more than 3 cm or in high-risk locations (near large blood vessels, gallbladder, bile ducts, gastrointestinal tract, or diaphragm) [5–7]. Moreover, incomplete RFA (iRFA) may accelerate the progression of residual tumors and hinder the programmed cell death protein-1 (PD-1) immunotherapy for malignant solid tumors [8–10]. Thus, it is clinically urgent to develop an effective treatment strategy to eradicate the residual tumors following RFA of malignant solid tumors.

After iRFA of malignant solid tumors, the exposed tumor antigens can be phagocytosed by dendritic cells (DCs) and presented to CD8^+^T cells, thereby activating an anti-cancer immune response. However, this anti-cancer effect is often limited in inhibiting the progression of the residual viable tumors [11, 12]. Therefore, inducing a strong anti-tumor immune response against the residual tumors may help lower the tumor recurrence rate after RFA of malignant solid tumors.

LTX-315, a first-in-class oncolytic peptide, can directly kill cancer cells by a membranolytic effect on the intracellular organelles and cellular plasma membrane after intratumoral injection, which further leads to a subsequent release of danger-associated molecular pattern molecules related to an immunogenic cell death (ICD) [13, 14]. Prior studies [14, 15] reported that LTX-315 could reprogram the tumor microenvironment by depleting intratumoral regulatory T cells (Tregs) and increasing the number of polyfunctional T cells. Cytotoxic T lymphocyte antigen-4 (CTLA-4) is a receptor expressed by T cells and can mediate opposite functions in T cell activation, thus inhibiting the anti-tumor immune function of T cells [16–18]. Meanwhile, previous studies also demonstrated that hyperthermia could enhance the efficacy of immunotherapy on malignant solid tumors by eliciting danger signals through the heat shock proteins, elevating the concentrations of immunotherapeutic agents in tumor cells, and reducing the resistance of tumors to the therapeutic agents [8, 14, 15, 19]. Given these findings, we were led to hypothesize that the combined therapy of LTX-315 with CTLA-4 inhibitors may lead to an optimal synergistic anti-tumor effect on residual tumors after RFA of malignant solid tumors, and may significantly reduce the tumor post-RFA recurrence rate of malignant solid tumors.

To the best of our knowledge, the anti-tumor immune mechanism of LTX-315 is still unclear. Presumably, the membranolytic effect of LTX-315 on plasma membrane can lead to an accumulation of DNA in the cytoplasm, which is perceived by the DNA sensors (cyclic guanosine phosphoadenosine synthase, cGAS) in cells, thereby activating the downstream protein of stimulator of interferon genes (STING), and causing secretion of type I interferon (IFN-I), resulting in an enhanced anti-tumor immunity [20–22].

In this study, we presented a combination treatment, that integrated RFA, LTX-315, and anti-CTLA-4 antibody, for more effective management of HCC, and explored the potential mechanisms, with an aim to reduce the tumor recurrence after RFA of malignant solid tumors and improve the long-term survival of these patients.

## Materials and methods

### Study design

This study consisted of two phases: (1) in vitro exploration of the enhancing effect of LTX-315 plus an anti-CTLA-4 antibody on the cytotoxicity of CD8^+^T cells for HCC cells after iRFA of HCC and the underlying mechanisms, and (2) in vivo validation of the feasibility of using LTX-315 plus an anti-CTLA-4 antibody for suppressing residual tumors after iRFA of HCC and the relevant mechanisms.

### In vitro experiments

#### Cell lines

Murine HCC Hepa1-6 cell line (Chinese Academy of Sciences, Shanghai, China) was authenticated by short tandem repeats profiling, and the experiments were conducted with mycoplasma-free cells. The Hepa1-6 cells were cultured in Dulbecco’s Modified Eagle Medium (Gibco), supplemented with 10% fetal bovine serum (Gibco), and maintained in an incubator at 37 °C with 5% CO_2_. Each in vitro cell experiment was repeated three times for statistical analysis.

#### Induction and extraction of CD8^+^ T cells from residual Hepa1-6 tumors after iRFA

Hepa1-6 cells (1 × 10^7^) were injected into the thigh muscle of C57BL/6 mice (male, 6–8 weeks old, Changsheng Biotechnology Co., Ltd, Liaoning, China), and tumor progression was ultrasonographically monitored. Once the tumor diameter reached ~1 cm, the tumor tissues were harvested, and then cut into small fragments sized about 1-mm^3^ in Hanks’ solution. A small incision was then made in the capsule of left liver lobe, and the small tumor fragment was implanted. Subsequently, small gelatin sponge strips were inserted into the incision to control bleeding, followed by suturing of the abdominal incision. The growth of the intrahepatic tumor was monitored by ultrasound imaging every 7 days. The treatments were initiated when the tumor diameter arrived at 6–8 mm.

The tumor-bearing mice were subjected to RFA treatment by using a 17-gauge single ablation electrode (RITA Medical Systems, Inc., Mountain View, CA, USA) under general anesthesia with 1–3% isoflurane (Piramal Healthcare, Andhra Pradesh, India). To create an incomplete tumor ablation model of orthotopic HCC in mice, the electrode was carefully inserted into the tumor from its left edge under ultrasound guidance. Ablation was then carried out at a power output of 70 °C, lasting for 2 min, which caused necrosis of ~70% of tumor cells and left about 30% of tumor cells viable.

The Hepa1-6 tumor-bearing mice were then divided into three groups (with 8 in each group):(1) control group: the liver tumors were treated by the ablation electrode puncture alone; (2) iRFA group: the liver tumors received iRFA as described in the ablation protocol; (3) iRFA + LTX-315 group: the liver tumors were subjected to iRFA, then LTX-315 (0.5 mg) was injected into the residual tumor. The LTX-315 administration to the residual tumors was repeated 3 days later under ultrasound guidance. One week later, CD8^+^ T cells in the residue viable tumors were extracted from each group by using a CD8^+^ T Cell Isolation Kit (EasySep, BD).

#### Treatments of Hepa1-6 cells

The CD8^+^T cells (1 × 10^5^/group) extracted from the aforementioned three groups were divided into seven groups and co-cultured with an anti-CTLA-4 antibody (10 μg/mL, Bio X Cell, New Hampshire) and Hepa1-6 cells (1 × 10^6^/group) in various combinations for 48 h. In terms of the combinations, the groups were designated (1) group A: the Hepa1-6 cells were cultured separately; (2) group B: the CD8^+^T cells from the control group were co-cultured with Hepa1-6 cells; (3) group C: the CD8^+^T cells from the control group were co-cultured with Hepa1-6 cells and the anti-CTLA-4 antibody; (4) group D: the CD8^+^T cells from the iRFA group were co-cultured with Hepa1-6 cells; (5) group E: the CD8^+^T cells from the iRFA group were co-cultured with Hepa1-6 cells and the anti-CTLA-4 antibody; (6) group F: the CD8^+^T cells from the iRFA-plus-LTX-315 group were co-cultured with Hepa1-6 cells; (7) group G: the CD8^+^T cells from the iRFA-plus-LTX-315 group were co-cultured with Hepa1-6 cells and the anti-CTLA-4 antibody.

#### Flow cytometrical analysis of CD8^+^T cells in the treated tumors and treated Hepa1-6 cells

To examine the expression of PD-1 and CTLA-4 in CD8^+^T cells in the control group, iRFA group, and iRFA + LTX-315 group, the CD8^+^T cells in treated tumors of three groups were collected by using a CD8^+^T Cell Isolation Kit (EasySep, BD), the antibodies to PD-1 and CTLA-4 were subsequently added, and then the expression levels were cytometrically analyzed.

To study the apoptosis of Hepa1-6 cells in groups A–G at 48 h after treatments, the Hepa1-6 cells were taken after washing three times with phosphate-buffered saline (PBS), Annexin-V (eBioscience, California) and PI (eBioscience, California) were subsequently added to the Hepa1-6 cells, and then the flow cytometry was performed on a fluorescence-activated cell sorting (FACS) flow cytometer (Canto II, BD). The data were analyzed by using a FlowJo software package (Treestar).

#### Cytotoxicity assay of in vivo-induced tumor-specific CD8^+^T cells

ELISPOT assays were conducted to evaluate the cytokine production by tumor-specific CD8^+^ T cells. Briefly, CD8^+^ T cells from the aforementioned groups were isolated using a CD8^+^ T Cell Isolation Kit, and 10,000 cells per group were plated in 96-well plates, that is coated with specific capture antibodies to IFN-γ (BD Biosciences, San Diego, CA, USA). After incubation, the cells were washed, and the biotinylated detection antibodies were added. Following further incubation, streptavidin-HRP was introduced, and spots were visualized using a substrate solution (AEC, Sigma-Aldrich). The number of spots, indicative of cytokine-producing cells, was quantified using an automated ELISPOT reader (Autoimmun Diagnostika GmbH, Germany). The results were expressed as spot-forming units per million cells. Hepa1-6 cells (1 × 10^4^) were plated into each well of a 96-well plate and treated as aforementioned, and the cell counting kit-8 (CCK-8) assay was used to evaluate the viability of treated Hepa1-6 cells. Besides, Hepa1-6 cells (1 × 106) were plated into each well of a 6-well plate and treated as aforementioned. The flow cytometry was conducted to assess the apoptosis of treated Hepa1-6 cells.

#### Hepa1-6 cells purification

Hepa1-6 cells were isolated from a co-culture of Hepa1-6 and CD8^+^ T cells using the Magnetic Cell Separation System from Miltenyi Biotec (Cat. No. 130-090-862), following the manufacturer’s instructions. Briefly, a mixture of Hepa1-6 and CD8+ T cells was incubated with CD8 T Cell Isolation Kit, Human (Miltenyi Biotec, Cat. No. 130-096-495), which was conjugated with magnetic microbeads targeting the CD8+ T cell surface markers. After incubation, the cells were passed through a magnetic column to retain the CD8+ T cells, while the non-labeled tumor cells were collected as the purified population for downstream applications.

#### Western blot assay of treated Hepa1-6 cells

The treated Hepa1-6 cells in the seven groups were harvested. Protein extracts were prepared on ice using a cold Radio-Immunoprecipitation Assay (RIPA) Lysis buffer. The proteins (20 μg) were then subjected to 8% sodium dodecyl sulfate-polyacrylamide gel electrophoresis (SDS-PAGE) and transferred onto PVDF membranes (Millipore, Billerica, MA, USA). The signals were detected by Western blotting using primary antibodies (Supplementary Table 1), and then by the corresponding peroxidase-conjugated secondary antibodies and Immobilion Western Chemilum HRP Substrate (Millipore, Billerica, MA, USA).

#### Quantitative real-time polymerase chain reaction (qRT-PCR) and enzyme-linked immunosorbent assay (ELISA) of treated Hepa1-6 cells

The total RNA from the seven groups of Hepa1-6 cells was extracted with a Qiagen RNeasy Mini Kit, and a cDNA was synthesized using the iScript™ Reverse Transcription Supermix (Bio-Rad, Hercules, CA). All primer sequences are provided in Supplementary Table 2. Subsequently, the mRNA levels of the downstream cytokines of the cGAS-STING pathway (TNF-α, CXCL9, CXCL10, and IFN-β) were measured by qRT-PCR on the ABI ViiA™ 7 Real-Time PCR System (Applied Biosystems). The housekeeping gene β-actin was used as an internal control. Relative quantification was performed by using the comparative Cycle Threshold (CT) method by following the manufacturer’s instructions. The concentrations of IFN-β, TNF-α, CXCL9, and CXCL10 in the cell culture media of the seven groups were measured by ELISA using high-sensitivity ELISA kits (eBiosciences, California).

### In vivo experiments

#### Establishment of an orthotopic HCC model in mice

The orthotopic live cancer model of mice was established as mentioned above, and the tumor growth was ultrasonographically observed every 7 days. Once the tumor grew to a size of 6–8 mm in diameter, mice were given the corresponding treatments. All animal experiments conducted in this study were approved by the Animal Care and Use Committee of our institution ([2024] IACUC Number: 3831).

#### The treatments of murine orthotopic HCC

In this study, all animals used were C57BL/6 mice (6–8 weeks old, male). Inclusion criteria for the animals were: healthy, no disease symptoms, no significant weight loss, and no noticeable behavioral abnormalities. Exclusion criteria included: failure to establish the tumor model (e.g., tumors not successfully implanted), and early mortality due to health issues (e.g., significant weight loss or other clinical symptoms). The included tumor-bearing animals were randomly assigned into the following five groups (*n* = 6/group): (1) control group: tumors received pseudo-RFA with a peritumoral injection of PBS (100-μL). Pseudo iRFA refers to the ablation electrode being only advanced into the tumor without ablation treatment; (2) iRFA group: tumors received iRFA treatment alone; (3) iRFA + LTX-315 group: tumors were subjected to iRFA treatment, followed by peritumoral injection of LTX-315 (0.5 mg, MedChemExpress, New Jersey) in 100-μL PBS; (4) iRFA + anti-CTLA-4 antibody group: tumors received iRFA, followed by intraperitoneal injection of an anti-CTLA-4 antibody (100 μg, Bioxcell, USA) in 100-μL PBS; (5) iRFA + LTX-315 + anti-CTLA-4 antibody group: tumors underwent iRFA, followed by peritumoral injection of LTX-315 and intraperitoneal injection of an anti-CTLA-4 antibody. Randomization was performed using simple randomization. The peritumoral injection of LTX-315 was repeated on days 3, 6, and 9 after the initial treatment under ultrasound guidance, and the intraperitoneal injection of an anti-CTLA-4 antibody was also repeated at the same time points. The iRFA procedures were conducted as aforementioned.

#### The assessment of tumor growth and mice survival

The tumor size was measured every 7 days under ultrasound guidance and calculated according to the following equation: *v* = *x*·*y*·*z*·*π*/6 (where *x* is longitudinal diameter, *y* axial diameter, and *z* depth diameter). The survival of mice in the five groups was recorded. The survival of mice in each group was followed up for 50 days from the day of treatment. Mice were euthanized if the maximum tumor diameter exceeded 2 cm or if they displayed signs of morbidity within the observation period. The time of euthanasia was recorded as the time of mortality.

#### Assessment of abscopal effect and rechallenge test

For the assessment of the abscopal effect, Hepa1-6 cells (1 × 10^6^) were subcutaneously injected into the right thigh of orthotopic Hepa1-6-bearing mice. The tumor size of the subcutaneous tumor was measured every 7 days under ultrasound guidance. With the rechallenge test, the corresponding treatments against orthotopic tumors were discontinued after 9 days of treatments. The age-matched healthy mice, that survived for 23 days after initial treatments, were injected subcutaneously with Hepa1-6 cells (1 × 10^6^ in 100 μL PBS) into the right thigh. The size of subcutaneous tumors was recorded by ultrasound imaging every 7 days, and the tumor volume was calculated as described above.

#### Histopathological analysis

After the euthanasia of mice, the tumors 14 days after treatments were harvested, fixed in 4% paraformaldehyde, paraffin-embedded, and sectioned at a thickness of 4 μm. For each tumor, six sections were histopathologically examined. Tissue sections from the heart, lung, kidney, and spleen were subjected to hematoxylin and eosin (H&E) staining. Tumor tissue sections were analyzed by H&E, CD4, CD8, NK-p44, Foxp3, terminal deoxynucleotidyl transferase dUTP nick end labeling (TUNEL), Ki-67, PD-1, and CTLA-4 staining. The Integrated Optical Density (IOD) sum of images or the percentages of CD8, CD4, Foxp3, NK-p44, TUNEL, Ki-67, PD-1, and CTLA-4 positive cells were automatically calculated by Image J software (National Institutes of Health) in five fields that contained the highest number of positively expressing cells. Results from these five areas were averaged and employed for the subsequent statistical analysis.

#### Western blotting, qRT-PCR, and flow cytometry of treated tumors

For quantitative analysis of the activation of ICD and cGAS-STING pathway in the treated tumors, the tumors were lysed in RIPA buffer containing protease and phosphatase inhibitors prior to SDS-PAGE and Western blotting. For the qRT-PCR of treated tumors, the total RNA was extracted from the tumor tissues using TRIzol (Invitrogen) and homogenized by following the manufacturer’s instructions. For the flow cytometry of treated tumors, the tumors were harvested, homogenized, and digested with a collagenase and hyaluronidase solution. The suspension was filtered through a cell mesh and resuspended in Hank’s media supplemented with 1% fetal calf serum for further analysis. Antibodies (eBioscience, California) against CD45, CD3, CD4, CD8, IFN-γ, TNF-α, NKR-P1A, Foxp3, CD44, CD80, CD86, GR1, CD11b, F4/80, CD206 were used. The production of IFN-gamma and TNF-alpha by tumor-infiltrating CD8+ T cells was determined through intracellular cytokine staining. The cells were stimulated in vitro with a non-specific activation method, such as phorbol myristate acetate and ionomycin, which are commonly used to induce cytokine production in T cells. Following the stimulation, cells were fixed and permeabilized to allow intracellular staining for IFN-gamma and TNF-alpha. These cytokines were then detected using specific antibodies against IFN-gamma and TNF-alpha (eBioscience). Subsequently, the flow cytometry was conducted on a FACS flow cytometer (Canto II, BD), and the data were analyzed by utilizing FlowJo software (Treestar, Oregon).

#### Validation of combination therapy efficacy after silencing cGAS-STING pathway and ICD-mediated immune effects

CRISPR-Cas9 was used to knock out cGAS and STING genes in Hepa1-6 cells. Cells were transfected with CRISPR plasmids targeting cGAS and STING (Addgene, USA) (cGAS guide RNA: 5′-GCTTCAATCTTGAGAGTGGG-3′; STING guide RNA: 5′-GAGAGGAGTGT GAGGAGGAG-3′) using Lipofectamine™ 3000 (Invitrogen, CA, USA). The knockout efficiency was confirmed by Western blotting. After gene knockout, six mice per group were obtained using the aforementioned tumor model method, and treatment was administered according to the above grouping and treatment protocol. CD11c monoclonal antibodies (BioXCell, NH, USA) were administered intraperitoneally at 100 μg/mouse, starting 7 days before combination therapy and repeated weekly for 3 weeks.

#### ELISA

The blood of mice was collected via the tail vein into tubes containing Ethylene Diamine Tetraacetic Acid. The plasma was obtained by centrifugation at 1000 g for 10 min and stored at −80 °C until analysis. The concentrations of TNF-α, CXCL9, CXCL10, and IFN-β in the plasma were measured by ELISA.

The peripheral blood, spleen, and tumor samples were taken from the treated mice. The peripheral blood was coagulated for 30 min. The tumors and spleens were dispersed in PBS and centrifuged for 5 min at 1000 g to harvest the supernatant. The levels of TNF-α, IFN-γ, IL-10, and TGF-β in the supernatant were quantified by ELISA (Biosciences, California).

#### Safety assessment

The levels of glutamic pyruvic transaminase (ALT), glutamic oxaloacetic transaminase (AST), creatine kinase (CK), and creatinine (CREA) were analyzed 21 days after initial treatments. Additionally, normal liver, heart, spleen, lung, and kidney tissues were H&E-stained for the evaluation of treatment safety.

#### Statistical analysis

Statistical analysis was conducted using SPSS V.24.0 software (SPSS Inc, Chicago, IL) and GraphPad Prism (Version 8.0, GraphPad Software, San Diego, California). The quantitative data were expressed as mean ± standard deviation and were analyzed using the Kruskal–Walli’s test or one-way analysis of variance. The overall survival of treated mice among different groups were evaluated with the Kaplan–Meier method and compared by using the log-rank test. The sample size was chosen based on a power analysis conducted prior to the experiment to ensure adequate power (80%) to detect a pre-specified effect size. During the experiment, the investigator was blinded to the group allocation for both treatment and outcome assessment. This blinding was maintained throughout the entire experiment, including during tumor measurement and survival analysis. The extent of blinding ensured that the investigator did not have prior knowledge of the group assignments to prevent any bias in the results. A *p* value less than 0.05 was considered statistically significant.

## Results

### In vitro experiments

#### LTX-315 plus anti-CTLA-4 antibody enhanced the cytotoxicity of CD8^+^T cells on Hepa1-6 cells after iRFA

**Fig. 1:**
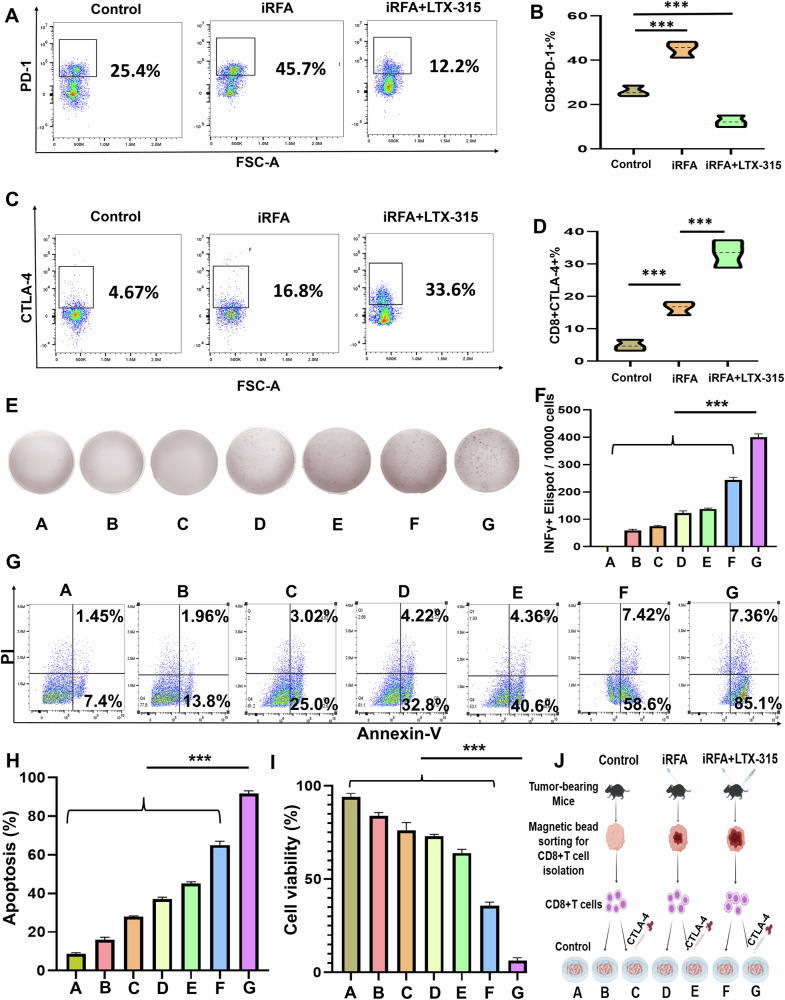
LTX-315 plus an anti-CTLA-4 antibody enhanced the cytotoxicity of CD8^+^T cells on Hepa1-6 cells after iRFA of Hepa1-6 tumors. **A**–**D** The flow cytometry was applied to analyze the expression of PD-1 and CTLA-4 on CD8^+^T cells in the residual tumors after iRFA or iRFA + LTX-315. **E**, **F** The ELISPOT assay was used to assess the proportion of IFN-γ^+^CD8^+^ T cells. **G**, **H** Flow cytometry (Annexin-V and PI) and quantitative analysis were used to measure the apoptosis rate in Hepa1-6 cells. **I** The CCK-8 assay was applied to evaluate the cell viability. **J** A schematic diagram explaining the grouping of A–G groups. *n* = 3 in each group. Error bars represent standard deviation. ***p < 0.001. CTLA-4 cytotoxic T lymphocyte antigen-4, iRFA incomplete radiofrequency ablation, PD-1 programmed cell death protein-1, ELISPOT enzyme-linked immunospot, CCK-8 cell counting kit-8.

The iRFA alone significantly increased the expression of PD-1 and CTLA-4 in CD8^+^T cells in the residual tumors. After additional treatment of LTX-315, PD-1 expression was significantly reduced and CTLA-4 expression was significantly elevated in CD8^+^T cells in the residual tumors (Fig. 1A–D). Moreover, the ELISPOT assay revealed that the combined therapy of LTX-315 plus an anti-CTLA-4 antibody significantly increased the proportion of INFγ^+^CD8^+^T cells compared with the other six groups (Fig. 1E, F). Meanwhile, LTX-315 plus an anti-CTLA-4 antibody caused the highest apoptosis of Hepa1-6 cells and the lowest cell viability of Hepa1-6 cells among the seven groups (Fig. 1H, I).

#### LTX-315 plus an anti-CTLA-4 antibody induced an ICD and activated cGAS-STING pathway in Hepa1-6 cells

**Fig. 2:**
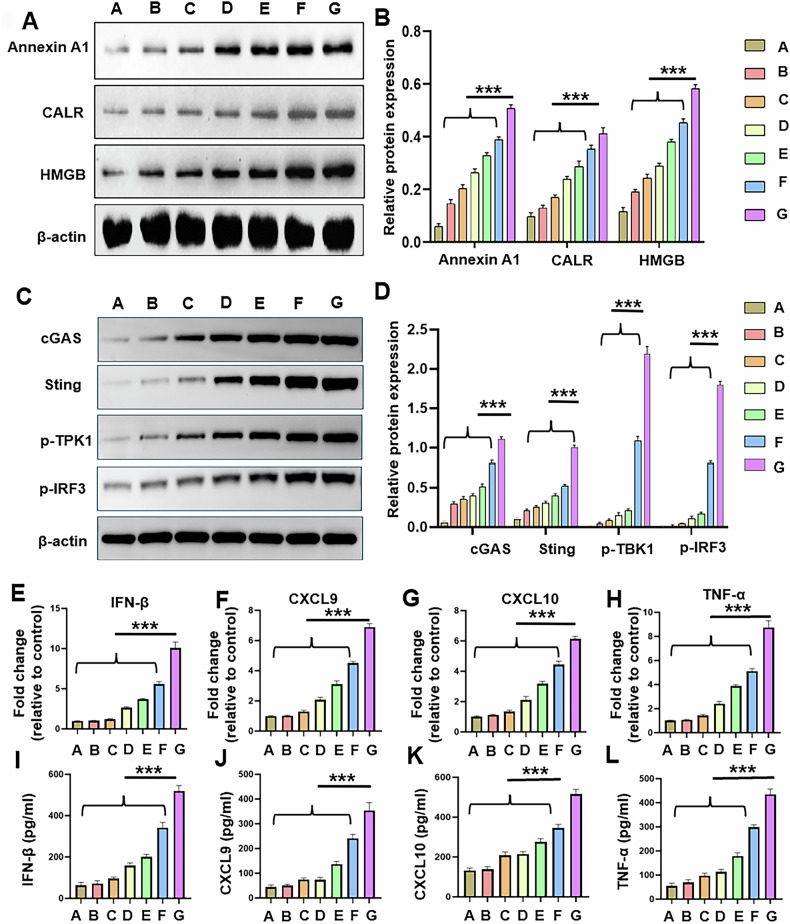
ICD and cGAS-STING pathway activation in the treated Hepa1-6 cells. **A**, **B** Western blotting was used to assess the expression of ICD proteins (ATP, CALR, HMGB1). **C**, **D** Western blotting was applied to measure the expression of cGAS-STING pathway proteins (cGAS, STING, p-TBK1, and p-IRF3). **E**–**H** qRT-PCR was used to measure the expression of TNF-α, CXCL9, CXCL10, and IFN-β. **I**–**L** ELISA was applied to evaluate the levels of TNF-α, CXCL9, CXCL10, and IFN-β in the cell culture medium. *n* = 3 in each group. Error bars represent standard deviation. ***p < 0.001. ICD immunogenic cell death, iRFA incomplete radiofrequency ablation, CTLA-4 cytotoxic T lymphocyte antigen-4, qRT-PCR quantitative real-time polymerase chain reaction, ELISA enzyme-linked immunosorbent assay.

LTX-315 in combination with an anti-CTLA-4 antibody induced significantly higher expression of ICD proteins (Annexin A1, CALR, HMGB) compared with the other six groups (Fig. 2A, B). Moreover, LTX-315 plus an anti-CTLA-4 antibody activated the cGAS-STING pathway by significantly up-regulating the expression of cGAS and STING proteins and significantly enhancing the phosphorylation of TBK1 and IRF3 in Hepa1-6 cells (Fig. 2C, D). Meanwhile, the expression of IFN-β, TNF-α, CXCL9, and CXCL10 in Hepa1-6 cells, and the secretion of IFN-β, TNF-α, CXCL9, and CXCL10 in the cell culture medium, were significantly higher in the group G than in the other six groups (Fig. 2E–L).

### In vivo experiments

#### Establishment of murine orthotopic HCC models

The orthotopic HCC was detected in the left liver lobe of mice 14 days after tumor implantation. The baseline tumor volume in the five groups was 122.4 mm^3^ ± 18.6, 119.5 mm^3^ ± 22.3, 121.7 mm^3^ ± 21.1, 124.7 mm^3^ ± 25.1 and 118.9 mm^3^ ± 19.5, respectively, with no statistically significant differences among them (*p* = 0.999).

#### Tumor response and survival of treated mice

**Fig. 3:**
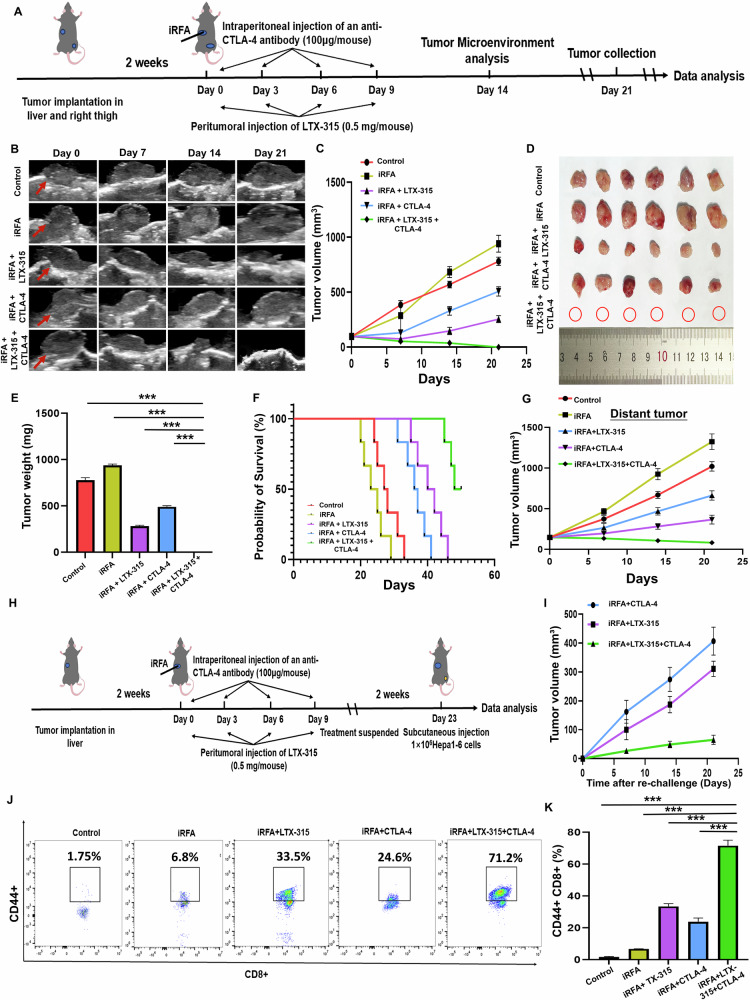
LTX-315 plus an anti-CTLA-4 antibody resulted in a strong anti-tumor effect on residue tumors after RFA of HCC. **A** Illustration of the study schedule for the treatment with the triple combination (iRFA + LTX-315 + anti-CTLA-4 antibody). **B**, **C** Tumor (red arrows) growth was observed by ultrasound imaging at days 0, 7, 14, and 21 after treatments. **D**, **E** Tumors were harvested from five groups to assess tumor size and weight. **F** Kaplan–Meier curves were generated to evaluate survival time. **G** Tumor growth in the right thigh of mice was measured. **H** Illustration of the study schedule for the rechallenge test for the triple combination treatment group. **I** The change of tumor volume in the rechallenge test. **J**, **K** Flow cytometry was used to measure the proportion of memory T cells (CD44^+^CD8^+^ T cells). *n* = 6 in each group. Error bars represent standard deviation. ***p < 0.001. CTLA-4 cytotoxic T lymphocyte antigen-4, iRFA incomplete radiofrequency ablation, HCC hepatocellular carcinoma.

As described in Fig. 3A, the ultrasound images of representative tumors in the five groups during follow-up are shown in Fig. 3B, and the corresponding tumor growth curves of the five groups are displayed in Fig. 3C, showing that the triple combination treatment group (iRFA + LTX-315+anti-CTLA-4 antibody) had the smallest tumor volume compared with the other four groups *(p* < 0.001). Twenty-one days after treatments, the liver tumors were harvested and weighted, and the results revealed that the tumor weight in the triple combination treatment group was the lowest among the five groups (*p* < 0.001) (Fig. 3D, E). A significantly prolonged survival time was observed in the triple combination treatment group compared with the other four groups (*p* < 0.001, Fig. 3F). Meanwhile, the abscopal effect test showed that the triple combination treatment inhibited more intensely the growth of extrahepatic tumors compared with the other four groups, indicating the triple combination treatment could induce a strong systematic immunity (Fig. 3G).

In the rechallenge test (as described in Fig. 3H), not enough mice survived to receive the rechallenge test in the control and iRFA-alone groups, and 6 mice in the iRFA + LTX-315 group, iRFA + CTLA-4 group, and iRFA + LTX-315 + CTLA-4 group were survived to receive the rechallenge test. The greatest inhibitory effect on the growth of rechallenged tumors was observed in the triple combination treatment group as compared with the other two groups (*p* < 0.001, Fig. 3I). The flow cytometrical results showed that the proportion of memory CD8^+^T cells (CD44^+^CD8^+^T cells) in the spleen was significantly higher in the triple combination treatment group than in the other four groups (*p* < 0.001, Fig. 3J, K). These results suggested that the triple combination treatment could induce a strong immunological memory.

#### LTX-315 plus an anti-CTLA-4 antibody activated the cGAS-STING pathway and induced an ICD after iRFA

**Fig. 4:**
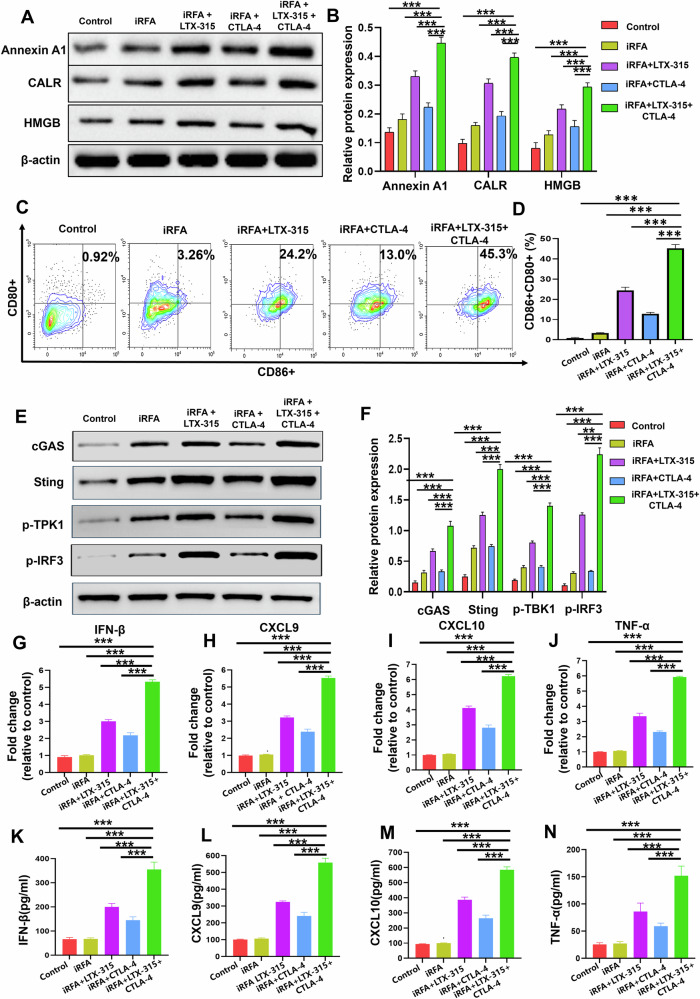
ICD and cGAS-STING pathway activation in residual tumors after treatments. **A**, **B** Western blotting was used to measure the expression of ICD proteins (ATP, CALR, HMGB1). **C**, **D** Flow cytometry was applied to assess the percentage of mature dendritic cells (CD80^+^CD86^+^ DC cells). **E**, **F** Western blotting was used to evaluate the expression of cGAS-STING pathway proteins (cGAS, STING, p-TBK1, and p-IRF3). **G**–**J** qRT-PCR was performed to measure the expression of TNF-α, CXCL9, CXCL10, and IFN-β in residual tumors. **K**–**N** ELISA was applied to assess the levels of TNF-α, CXCL9, CXCL10, and IFN-β in the mice plasma. *n* = 6 in each group. Error bars represent standard deviation. ***p < 0.001. ICD immunogenic cell death, iRFA incomplete radiofrequency ablation, CTLA-4 cytotoxic T lymphocyte antigen-4, qRT-PCR quantitative real-time polymerase chain reaction assay, ELISA enzyme-linked immunosorbent assay.

LTX-315 plus CTLA-4 antibody induced a significantly higher expression of ICD proteins (Annexin A1, CALR, HMGB) and a significantly higher percentage of mature DC cells (CD80^+^CD86^+^DC cells) compared with the other four groups (Fig. 4A–D). Furthermore, LTX-315 in combination with an anti-CTLA-4 antibody activated the cGAS-STING pathway by significantly increasing the expression of cGAS and STING proteins and significantly enhancing the phosphorylation of TBK1 and IRF3 in residual tumors (Fig. 4E, F). Additionally, the expression of IFN-β, TNF-α, CXCL9, and CXCL10 in residual tumors, and the secretion of IFN-β, TNF-α, CXCL9, and CXCL10 in the plasma, were significantly higher in the triple combination treatment group than in the other four groups (Fig. 4G–N).

#### LTX-315 plus an anti-CTLA-4 antibody elicited a strong anti-tumor immune response after iRFA

**Fig. 5:**
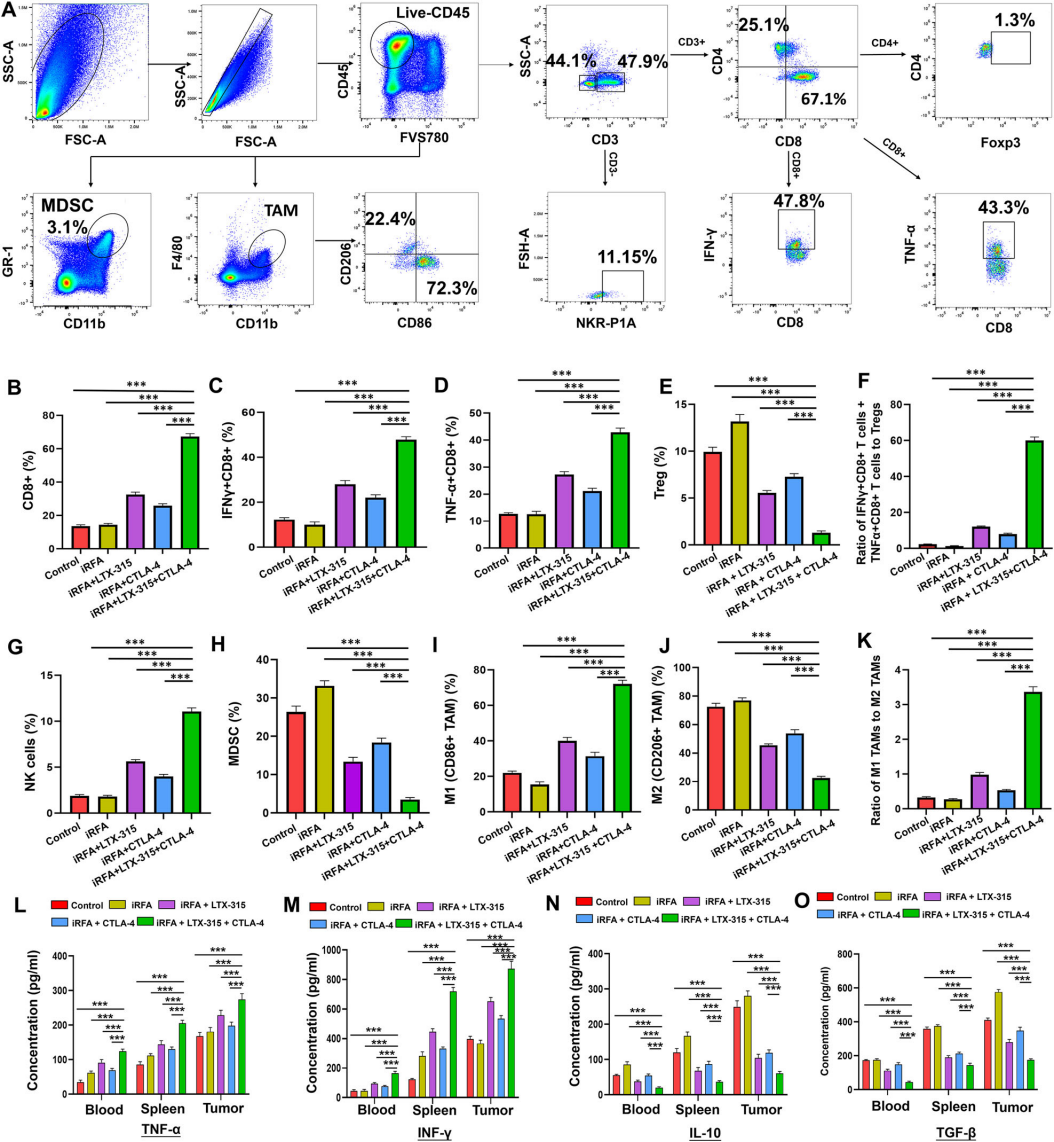
LTX-315 plus an anti-CTLA-4 antibody reprogrammed a strong anti-tumor immune microenvironment in residual tumors after iRFA of HCC. **A** Representative dot plots of tumors subjected to the triple combination treatment (iRFA + LTX-315 + anti-CTLA-4 antibody). Schematic illustration of gating: Immune cells in residual tumors, such as CD4^+^ T, CD8^+^ T, Tregs, TAMs, and MDSCs, were stained with the corresponding antibodies for flow cytometric analysis. **B**–**K** The percentages of CD8^+^ T cells, IFN-γ^+^CD8+ T cells, TNF-α^+^CD8^+^ T cells, Foxp3^+^CD4^+^ T cells, the ratio of IFN-γ^+^CD8^+^ T cells plus TNF-α^+^CD8^+^ T cells to Tregs, NK cells, MDSCs, and the ratio of M1-TAM to M2-TAM were assessed in tumors at 14 days after treatments. ELISA was applied to evaluate the levels of TNF-α (**L**), IFN-γ (**M**), IL-10 (**N**), and TGF-β (**O**) in the blood, spleen, and residual tumors. *n* = 6 in each group. Error bars represent standard deviation. ***p < 0.001. CTLA-4 cytotoxic T lymphocyte antigen-4, iRFA incomplete radiofrequency ablation, HCC hepatocellular carcinoma, Tregs regulatory T cells, TAMs tumor-associated macrophages, MDSCs myeloid-derived suppressor cells, ELISA enzyme-linked immunosorbent assay.

As shown in Fig. 5, the flow cytometry revealed a significant increase in the number of M1-like tumor-associated macrophages (M1-TAMs), CD8^+^T cells, TNFα^+^CD8^+^T cells, IFN-γ^+^CD8^+^T cells, and NK cells, a significant decrease of FoxP3^+^Tregs, myeloid-derived suppressor cells (MDSCs), and M2-like tumor-associated macrophages (M2-TAMs), a significantly higher ratio of functional CD8^+^ T cells (TNF-α^+^CD8^+^T cells and IFN-γ^+^CD8^+^T cells) to Tregs, and a significantly higher ratio of M1-TAMs to M2-TAMs in residual tumors in the triple combination treatment group than in the other four groups (Fig. 5). Meanwhile, the ELISA showed a significantly higher level of IFN-γ and TNF-α, and a significantly lower level of IL-10 and TGF-β in blood, spleen, and residual tumors in the triple combination treatment group than in the other four groups (Fig. 5L–O). These findings demonstrated that the combined therapy of LTX-315 with an anti-CTLA-4 antibody could induce a strong anti-tumor immune response after iRFA of HCC.

#### Impact of cGAS-STING pathway and ICD-mediated immune response silencing on tumor immunity and treatment efficacy

**Fig. 6:**
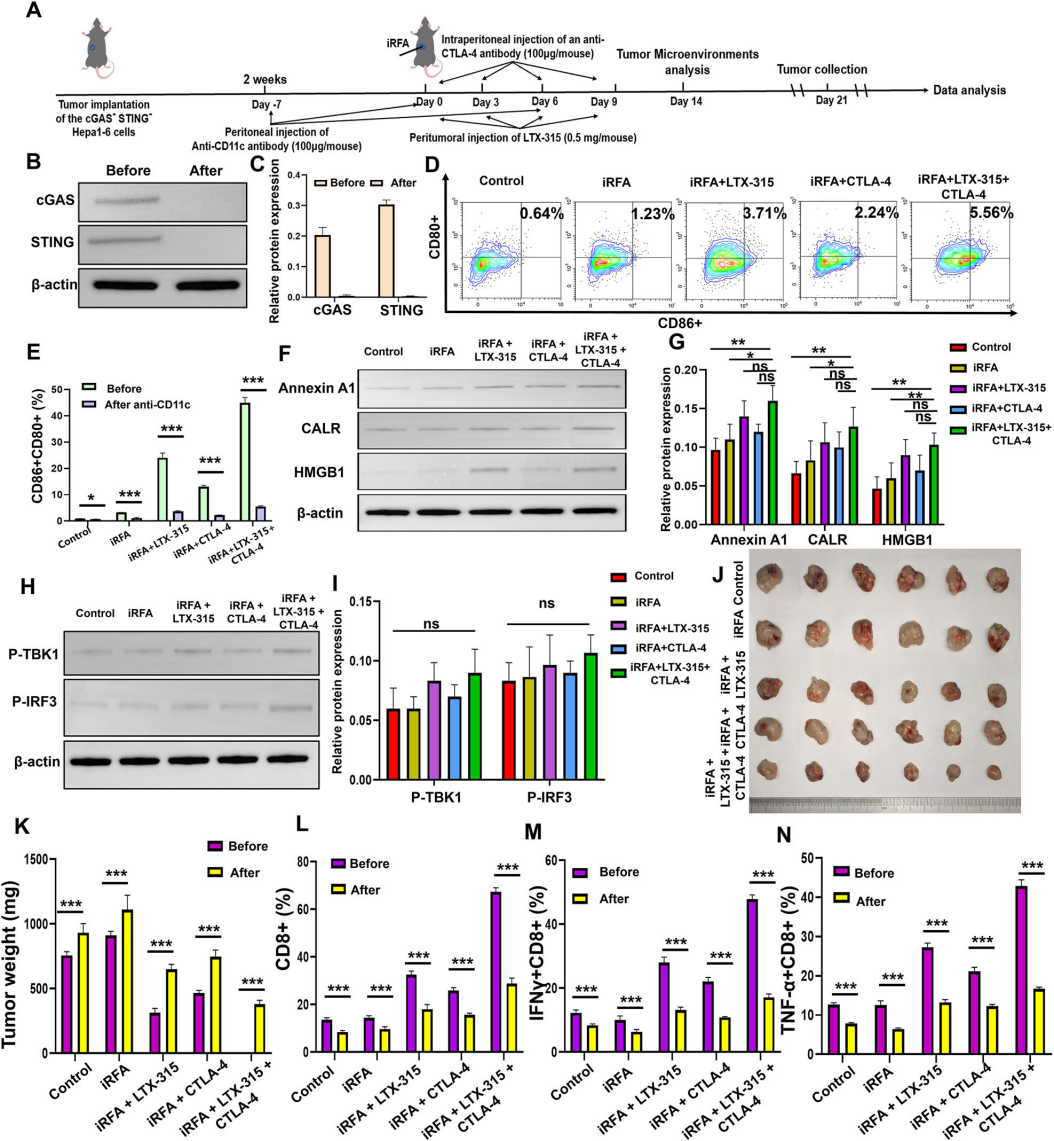
Silencing of cGAS-STING pathway and ICD-mediated immune response and its impact on tumor immunity. **A** Experimental workflow for silencing the cGAS-STING pathway and ICD in vivo studies. **B**, **C** Western blotting was used to confirm the knockout of cGAS and STING proteins in Hepa1-6 cells. **D**, **E** The proportion of mature dendritic cells (CD80^+^CD86^+^ DCs) was assessed after treatment with the anti-CD11c antibody. **F**, **G** Western blotting was applied to confirm the reduction of ICD-related molecules (ATP, CALR, HMGB1) after treatment with the anti-CD11c antibody. **H**, **I** Phosphorylation of TBK1 and IRF3 was reduced in tumors after silencing the cGAS-STING pathway. **J**, **K** Tumors were harvested and weight was assessed, showing an increase in tumor size after silencing the cGAS-STING pathway and ICD in all five groups. **L**–**N** Flow cytometry analysis was used to evaluate the levels of functional CD8^+^ T cells after silencing the cGAS-STING pathway and ICD in all five groups. *n* = 6 per group. Error bars represent standard deviation. Ns not statistically significant. *p < 0.05, **p < 0.01, ***p < 0.001.

Fig. 6A illustrates the experimental workflow for in vivo studies following the silencing of the cGAS-STING pathway and ICD-mediated immune response (By Figdraw, ID: IROYA1744). The results shown in Fig. 6B, C demonstrate successful knockout of the cGAS and STING molecules through Western Blot analysis, confirming the effective silencing of both targets. Figure 6D presents the proportion of mature DCs across the groups, and Fig. 6E provides a statistical chart showing a significant reduction in DC maturation after treatment with CD11c monoclonal antibody. Then the reduction of the maturated DC cells induced a marked decrease in ICD-related molecules within the tumor (Fig. 6F, G). In Fig. 6H, I, a clear reduction in the phosphorylation of TBK1 and IRF3 was observed, with no significant differences between the experimental groups, and the PCR and ELISA results of the downstream cytokine of the cGAS-STING pathway were shown in Supplementary Fig. 1. Figure 6J, K depicts the overall tumor appearance and weight statistics. Tumor weights were significantly heavier across all groups that underwent silencing of these molecules, in comparison to controls, suggesting an attenuation of anti-tumor immunity. Figure 6L–N shows a flow cytometry analysis of CD8+ T cells, revealing a substantial reduction in the number of functional CD8+ T cells, consistent with the disruption of immune response pathways in the treated groups, other changes in the immune cells were shown in Supplementary Fig. 2.

#### Histological examination

**Fig. 7:**
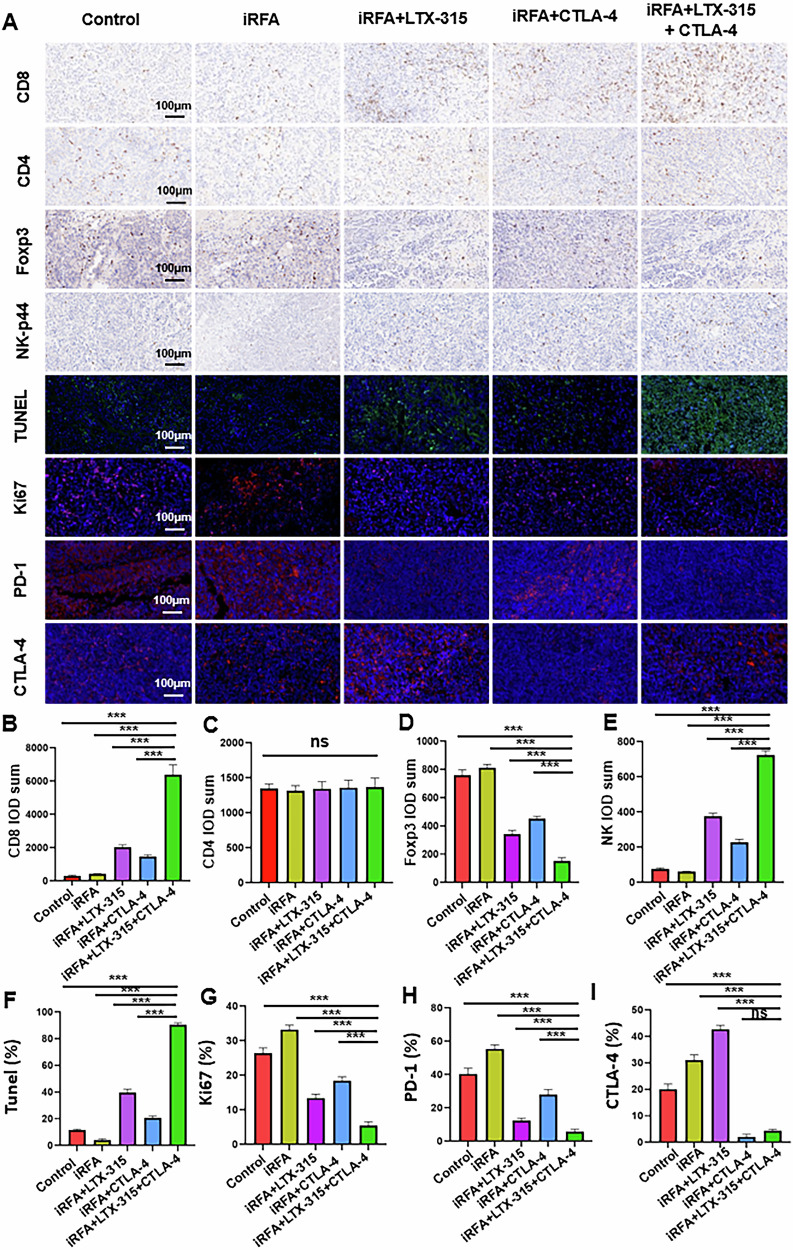
Pathological examination of the treated tumors in five groups. **A**, **B** CD8 staining was used to assess the presence of CD8^+^ T cells. **A**, **C** CD4 staining was used to evaluate the presence of CD4^+^ T cells. **A**, **D** NK-p44 staining was used to detect NK cells. **A**, **E** Foxp3 staining was applied to examine the presence of Tregs. **A**, **F**, **G** TUNEL and Ki-67 staining were used to evaluate apoptosis and proliferation of tumor cells. **A**, **H**, **I** PD-1 and CTLA-4 staining were used to assess the expression levels of PD-1 and CTLA-4 in tumors. *n* = 6 in each group. Error bars represent standard deviation. ns represents no statistically significant difference, ***p < 0.001. iRFA incomplete radiofrequency ablation, PD-1 programmed cell death protein-1, CTLA-4 cytotoxic T lymphocyte antigen-4.

The CD8 and CD4 staining showed that LTX-315 plus an anti-CTLA-4 antibody could significantly increase infiltration of the CD8^+^ T cells, but did not significantly enhance infiltration of the CD4^+^ T cells in residual tumors (Fig. 7A–C). The NK-p44 and Foxp3 staining exhibited that LTX-315 plus an anti-CTLA-4 antibody could significantly increase the NK cell infiltration, and significantly decrease the Tregs infiltration in the residual tumors (Fig. 7A, D, E). The TUNEL and Ki-67 staining showed that the triple combination treatment group exerted the strongest apoptotic effect on cells and the weakest proliferation-promoting effect on residual cells among the five groups (Fig. 7A, F, G). The PD-1 and CTLA-4 staining revealed that the iRFA could induce significantly higher expression of PD-1 and CTLA-4 in residual tumors, and additional LTX-315 led to a significant decrease of PD-1 and a significant increase of CTLA-4, which could be lowered by the anti-CTLA-4 treatment (Fig. 7H, I).

#### Safety assessment

**Fig. 8:**
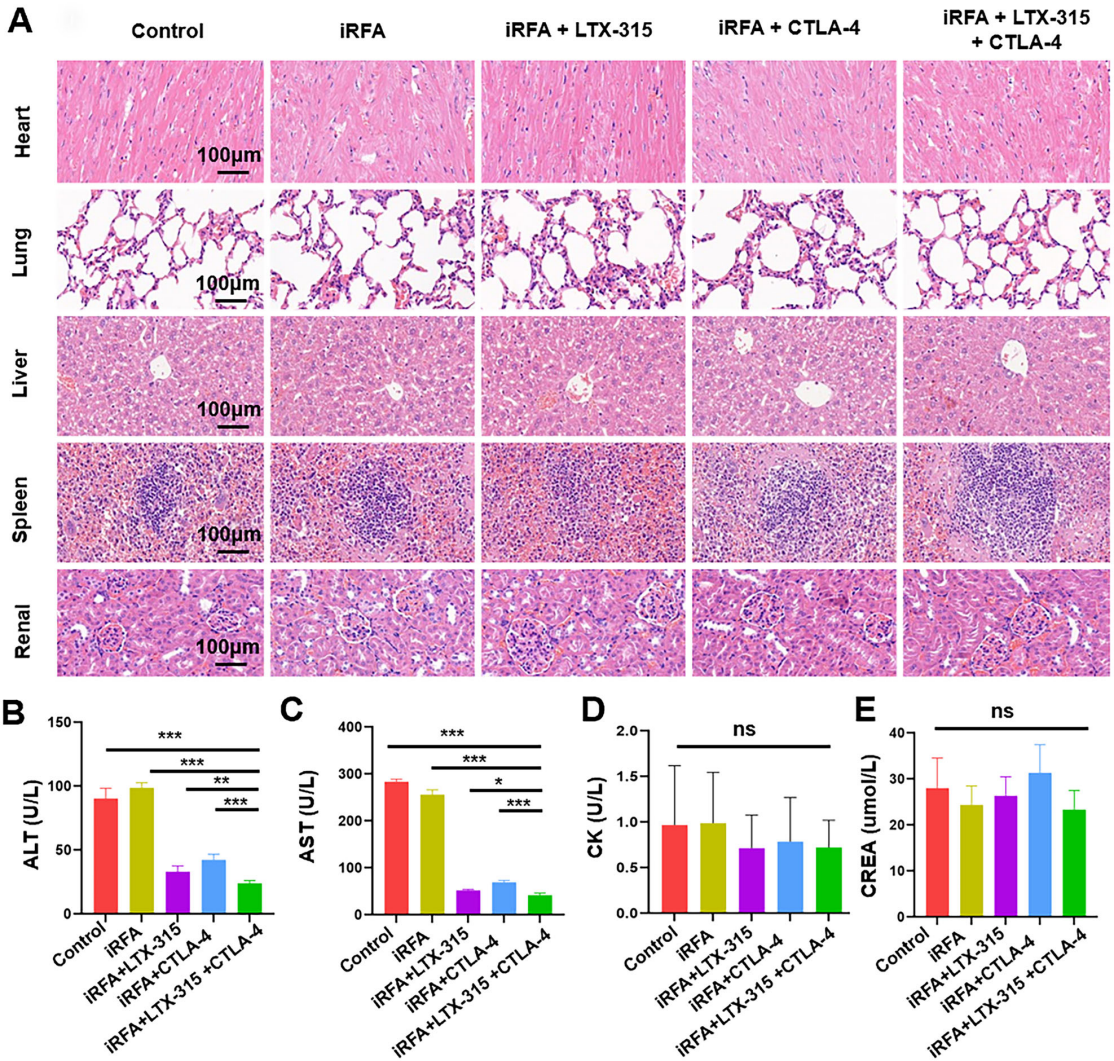
The safety assessment of the triple combination treatment (iRFA + LTX-315 + anti-CTLA-4 antibody) for HCC. **A** The typical HE-stained images of heart, liver, spleen, lung, and kidney tissues in five groups suggested that the triple combination treatment did not cause damage to the major organs. **B**–**E** Two liver function parameters, i.e., ALT and AST, were in the normal range in the triple combination treatment group, and were significantly lower than those in the control and iRFA groups. *n* = 6 in each group. Error bars represent standard deviation. ns represents no statistically significant difference. ***p < 0.001. iRFA incomplete radiofrequency ablation, CTLA-4 cytotoxic T lymphocyte antigen-4, HCC hepatocellular carcinoma, H&E Hematoxylin and eosin, ALT glutamic pyruvic transaminase, AST glutamic oxaloacetic transaminase.

H&E staining revealed no discernible pathological changes in the normal liver, heart, spleen, lung, and kidney of mice following the treatments in our study (Fig. 8A). ALT and AST, two liver function parameters, were in normal range and were significantly lower in the triple combination treatment group than in the control and iRFA groups (Fig. 8B, C). Meanwhile, the heart (CK) and kidney (CREA) functions of mice in the triple combination treatment group were normal (Fig. 8D, E).

## Discussion

Immunotherapy is a promising and efficacious treatment alternative for HCC. However, the tumor response rates remain relatively low due to some barriers [23, 24] such as an immunosuppressive microenvironment [25, 26] Present and previous studies [27, 28] showed the microenvironment of residual viable tumors after RFA of HCC is highly immunosuppressive, with the increased infiltration of immunosuppressive cells and the up-regulated expression of PD-1 and CTLA-4 in CD8^+^T cells. In this study, we found that LTX-315 significantly decreased the Tregs infiltration, significantly down-regulated the PD-1 expression, and significantly up-regulated CTLA-4 expression in residual tumors, which enhanced the tumor response to anti-CTLA-4 antibody treatment. The immunosuppressive microenvironment of residual tumors was significantly improved by the combined therapy of LTX-315 plus an anti-CTLA-4 antibody, as indicated by a high ratio of M1-TAMs to M2-TAMs, few Tregs and MDSCs, and lowered expression of PD-1 and CTLA-4. These results demonstrated that LTX-315 plus an anti-CTLA-4 antibody could synergistically improve the immunosuppressive microenvironment of residual tumors after iRFA of HCC.

Moreover, further activation of anti-tumor immunity could improve the therapeutic effect of RFA on HCC. In the present study, compared to the other four treatments, the triple combination treatment (iRFA + LTX-315 + anti-CTLA-4 antibody) significantly promoted the infiltration of CD8^+^T cells, NK cells, TNF-α^+^CD8^+^T cells, and IFN-γ^+^CD8^+^T cells, up-regulated the expression of TNF-α and INF-γ in blood, spleen, and residual tumors, and significantly raised the proportion of memory T cells (CD44^+^CD8^+^T cells) in the mice spleen, leading to an excellent tumor-inhibitory effect. These results indicated that the combined therapy of LTX-315 with an anti-CTLA-4 antibody could synergistically induce an intense anti-tumor immunity after iRFA of HCC.

To achieve a complete RFA of HCC, *a* > 5 mm ablative margin around the entire tumor must be created. However, to create such a safe margin is a great challenge for HCC with diameter >3 cm or in high-risk locations. In the present study, we attempted to address this critical problem by integrating the effects of radiofrequency hyperthermia, LTX- 315, and anti-CTLA-4 antibody. The results of our study showed that LTX-315 plus anti-CTLA-4 antibody yielded a favorable anti-tumor effect for residual tumors, which could help lower the tumor recurrence after iRFA of HCC.

On the basis of the results of this study, we were led to theorize that the mechanism of the triple combination treatment might be as follows: (1) The treatment could synergistically activate the cGAS-STING pathway in residual tumors after RFA of HCC, which can trigger a type I interferon response, subsequently leading to the recruitment and activation of various immune effector cells, including NK cells and cytotoxic T lymphocytes [29, 30]; (2) The treatment can jointly induce an ICD of the residual tumors, with an elevated expression of DAMPs, thereby promoting DC cells maturation, and subsequently triggering an adaptive anti-cancer immune response [31, 32]. As a result, the triple combination treatment in this study induced a strong anti-tumor immunity.

The safety of the triple combination treatment in this study was assessed by monitoring the serum ALT, AST, CK, CERA levels, and H&E staining of major organs. The results showed that the function of major organs was normal, and the liver function parameters ALT and AST were significantly lower in the triple combination treatment group than in the other four groups, which might be explained by less tumor burden in this group. These results demonstrated that LTX-315 plus anti-CTLA-4 antibody was safe for the treatment of residual tumors after iRFA of HCC.

In conclusion, the combined therapy of LTX-315 with an anti-CTLA-4 antibody is effective and safe for residual tumors after iRFA of HCC. LTX-315 plus an anti-CTLA-4 antibody could synergistically improve the immunosuppressive microenvironment in residual tumors and induce a strong anti-tumor immunity after iRFA of HCC. This combination treatment strategy may open a new avenue to reducing the residual and recurrent tumors after RFA of malignant solid tumors with large sizes or in high-risk locations and leading to further studies on its clinical applications.

## Supplementary information

Supplementary file

## Data Availability

The data of this study are available from the corresponding author on reasonable request.
