# Supplementary material for: Oncolytic peptide LTX-315 plus an anti-CTLA-4 antibody induces a synergistic anti-cancer immune response in residual tumors after radiofrequency ablation of hepatocellular carcinoma
Source: Cell Death Dis. 2025 Apr 13;16(1):288. doi: 10.1038/s41419-025-07622-z (PMC11994779; doi:10.1038/s41419-025-07622-z)

Supplementary Figure 1 Downstream cytokine levels of the cGAS-STING pathway significantly decreased after silencing cGAS-STING pathway. **A-D**: The qRT-PCR was used to measure the expression of TNF-α, CXCL9, CXCL10, and IFN-β in the treated tumors. **E-H：** ELISA was applied to measure the levels of TNF-α, CXCL9, CXCL10, and IFN-β in the plasma of the five groups. n=6 in each group. Error bars represent standard deviation. ns: no statistically significant difference.

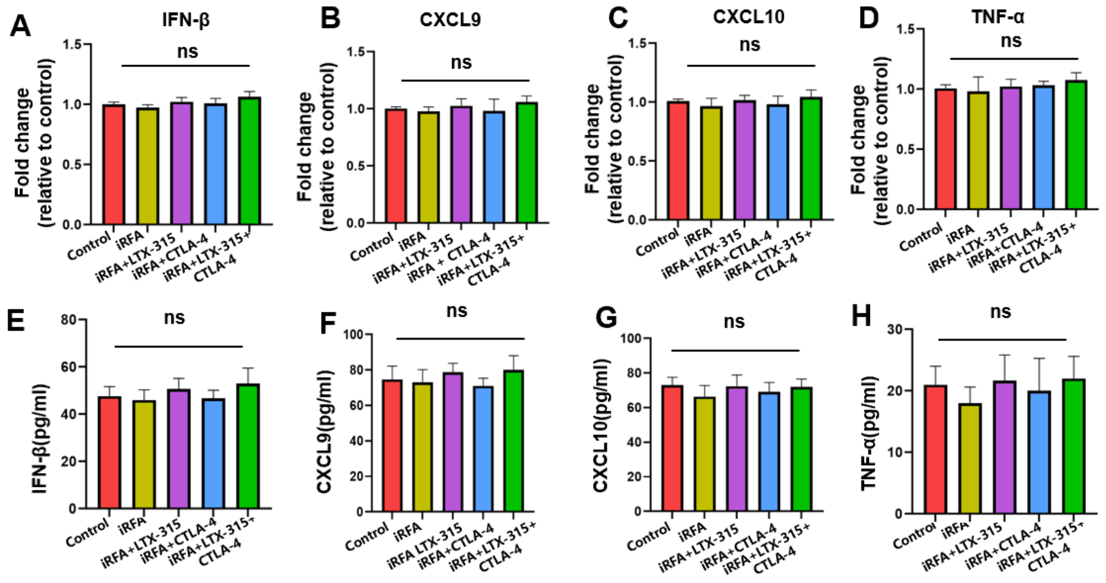

Supplementary Figure 2 The anti-tumor immune effect of combination therapy significantly decreased after silencing the cGAS-STING pathway and ICD-mediated immune response. **A:** Representative dot plots of the morphological characteristics (SSC vs FSC) of tumors subjected to the triple combination treatment (iRFA+LTX-315+anti-CTLA-4 antibody). Schematic illustration of gating: The immune cells in residual tumors, such as $CD4^+$ T, $CD8^+$ T, Tregs, TAMs, and MDSCs were stained with the corresponding antibodies for flow cytometric analysis. **B-G**: The quantitative analysis of the percentages of $Foxp3^+CD4^+$T cells, NK cells, MDSCs, M1-TAM, M2-TAM and the ratio of M1-TAM to M2-TAM in tumors at 14 days after treatments. n=6 in each group. Error bars represent standard deviation. ***$p < 0.001$. CTLA-4, cytotoxic T lymphocyte antigen-4; iRFA, incomplete radiofrequency ablation. Tregs, regulatory T cells; TAMs, tumor-associated macrophages; MDSCs, myeloid-derived suppressor cells;

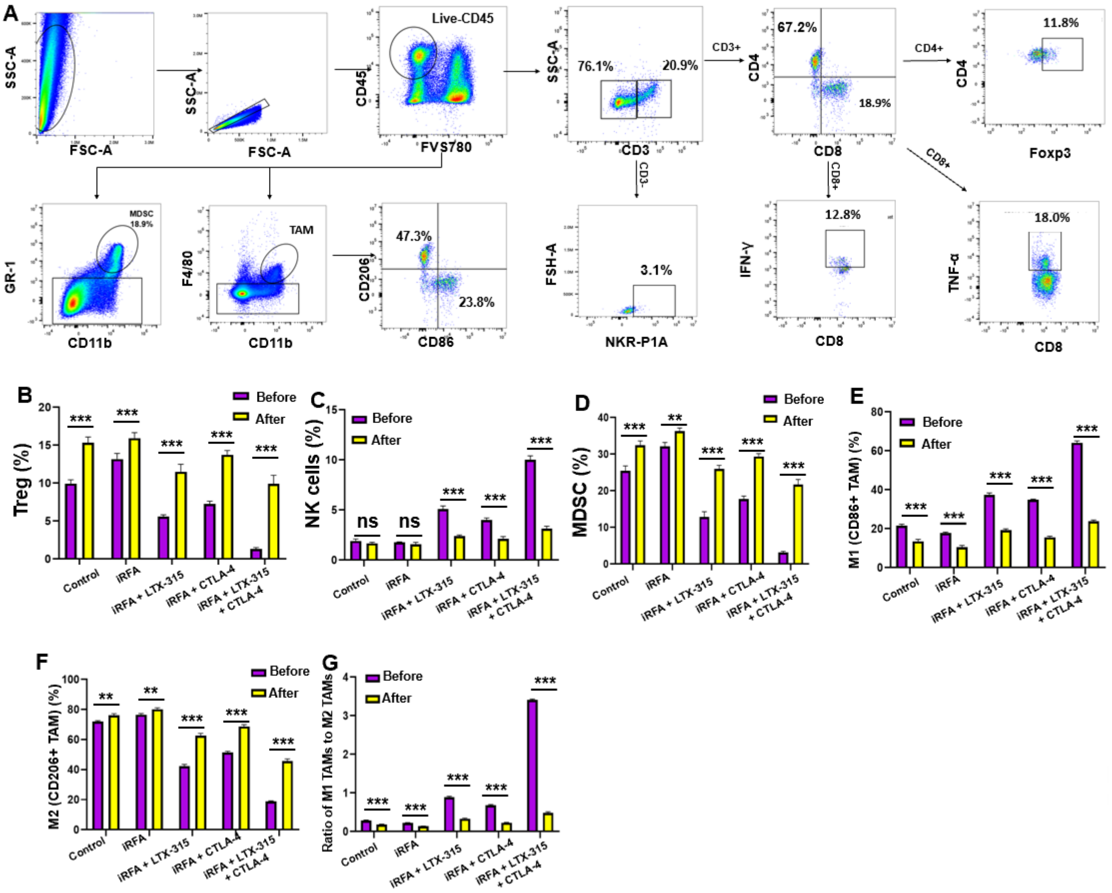

Supplemental Table 1: The antibodies used in western blotting.

	Primary antibodies					Secondary antibodies				
	Company	Item number	Molecular weight（KD）	Origen	Dilution ratio		Company	Item number	Dilution radio	Separation glue concentration
β-actin	Servicebio	GB15003-100	45	Rabbit	1: 3000	HRP Goat anti-mouse	Servicebio	GB25301	1：5000	10.0%
STING	Servicebio	GB111415	37	Rabbit	1: 1000	HRP Goat anti-rabbit	Servicebio	GB23303	1：5000	10.0%
cGAS	Proteintech	36416-1-AP	58	Rabbit	1: 1000	HRP Goat anti-rabbit	Servicebio	GB23303	1：5000	10.0%

P-TKB1	CST	5483	84	Rabbit	1: 1000	HRP Goat anti-rabbit	Servicebio	GB23303	1：5000	10.0%
P-IRF3	CST	29047	45-55	Rabbit	1: 1000	HRP Goat anti-rabbit	Servicebio	GB23303	1：5000	10.0%
Annexin A1	Servicebio	GB113305-100	34	Rabbit	1: 1000	HRP Goat anti-rabbit	Servicebio	GB23303	1：5000	10.0%
HMGB	Servicebio	GB11103-100	25	Rabbit	1: 1000	HRP Goat anti-rabbit	Servicebio	GB23303	1：5000	10.0%
CALR	Servicebio	GB112134-100	55	Rabbit	1: 1000	HRP Goat anti-rabbit	Servicebio	GB23303	1：5000	10.0%

Supplemental Table 2: Sequences of the primers for qPCR analysis.

Primers

Gene	Primers	Sequence (5’-3’)
TNF-α	Forward	CCCTCACACTCACAAACCACC
	Reverse	CTTTGAGATCCATGCCGTTG
CXCL9	Forward	TTTCCTCTTGGGCATCATCTTC
	Reverse	TAGTGGATCGTGCCTCGGCT
CXCL10	Forward	AAGCGTTTAGCCAAAAAAGGTC
	Reverse	ACTGGGTAAAGGGGAGTGATGG
IFN-β	Forward	CCTATGGAGATGACGGAGAAGATG
	Reverse	CAACAATAGTCTCATTCCACCCAG
β-actin	Forward	TGGAATCCTGTGGCATCCATGAAAC
	Reverse	TAAAACGCAGCTCAGTAACAGTCCG

WB

Figure 2A

ATP

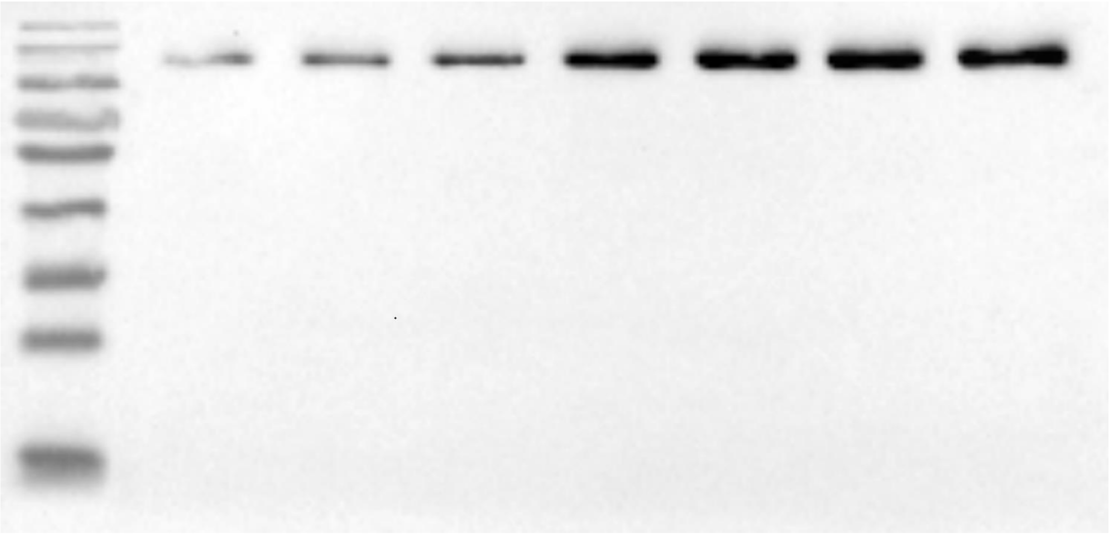

CALR

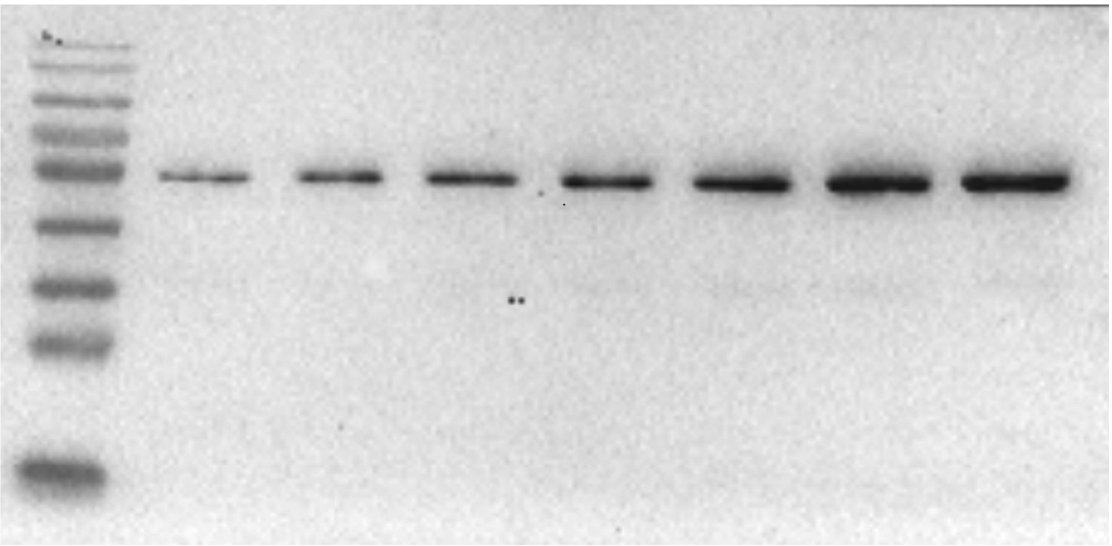

HMGB

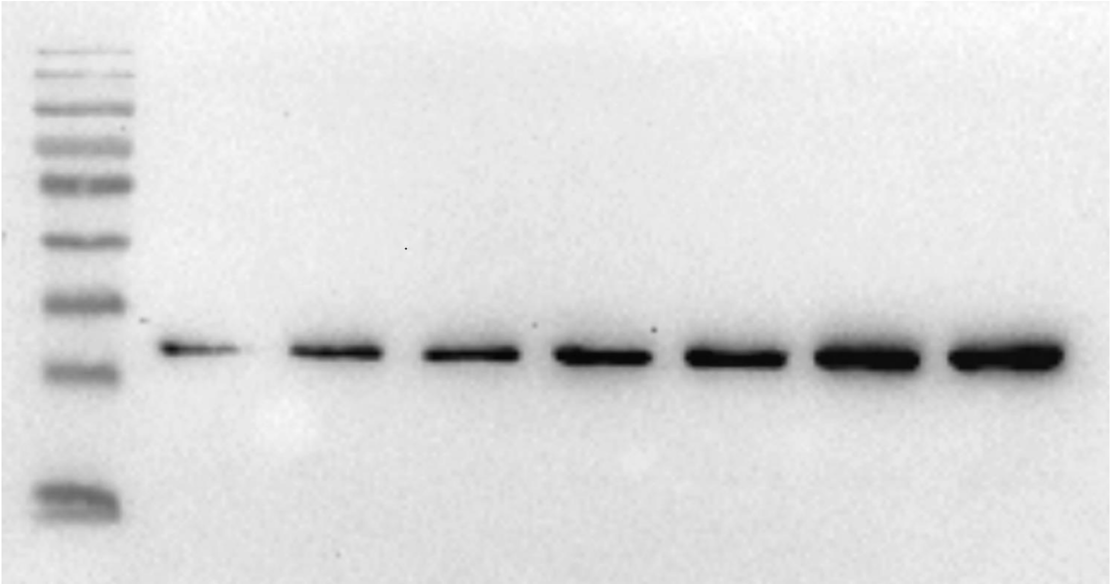

β-actin

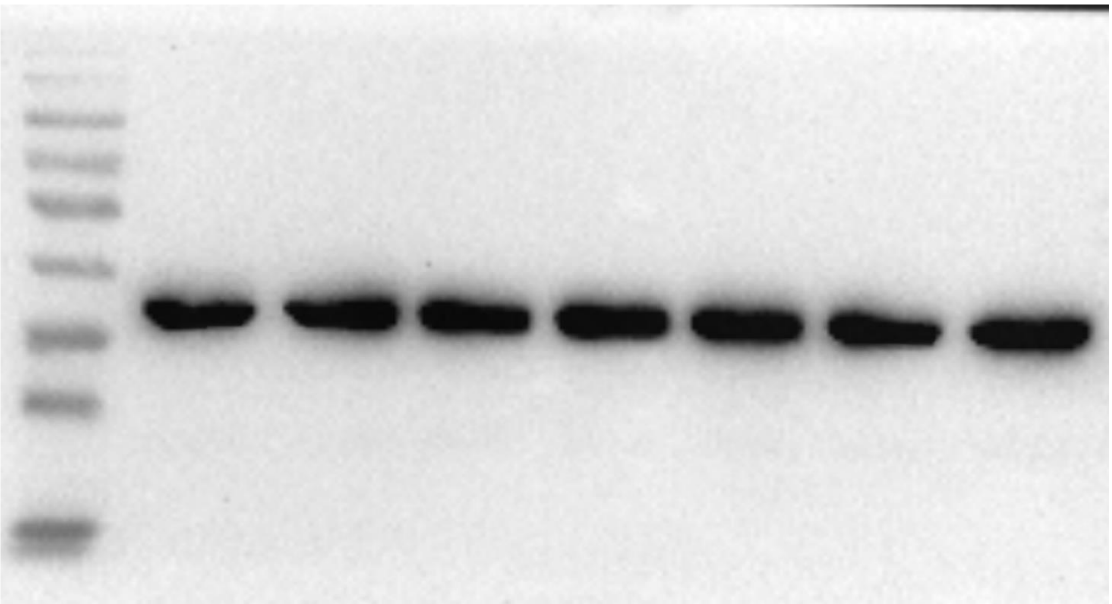

Figure 2C

cGAS

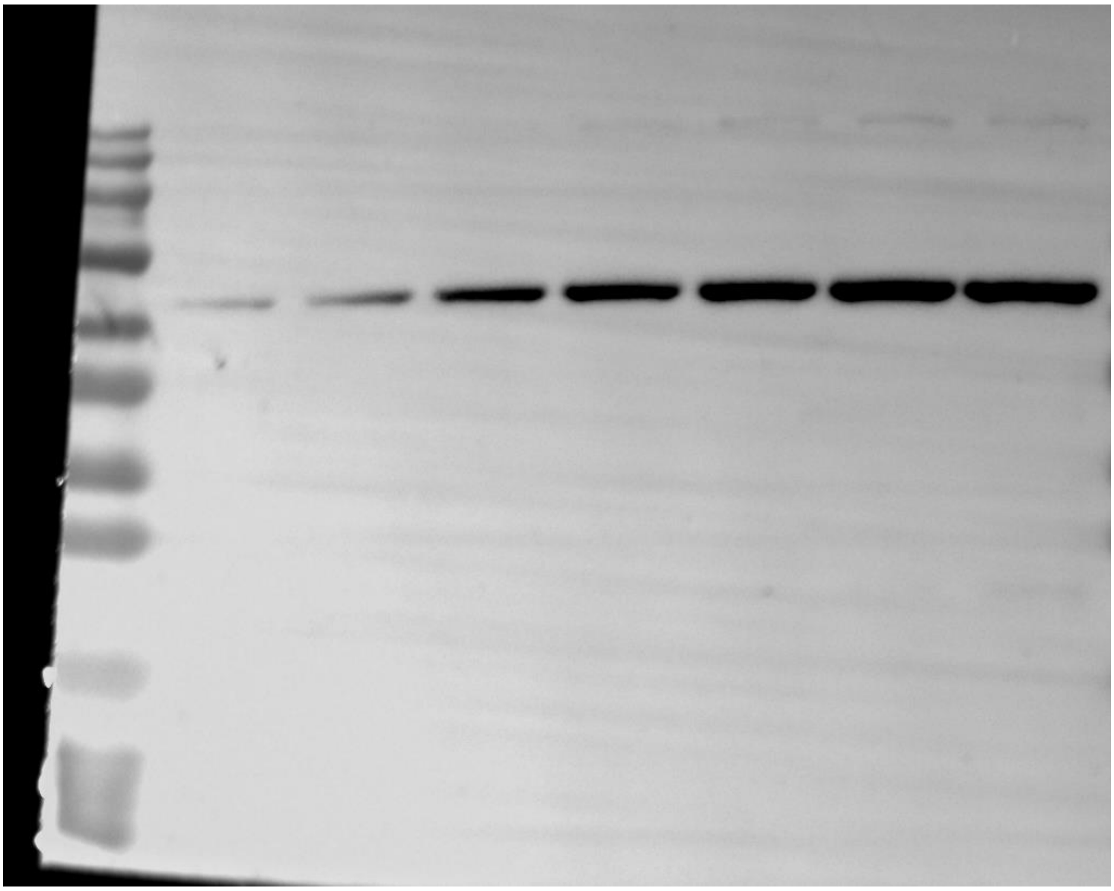

Sting

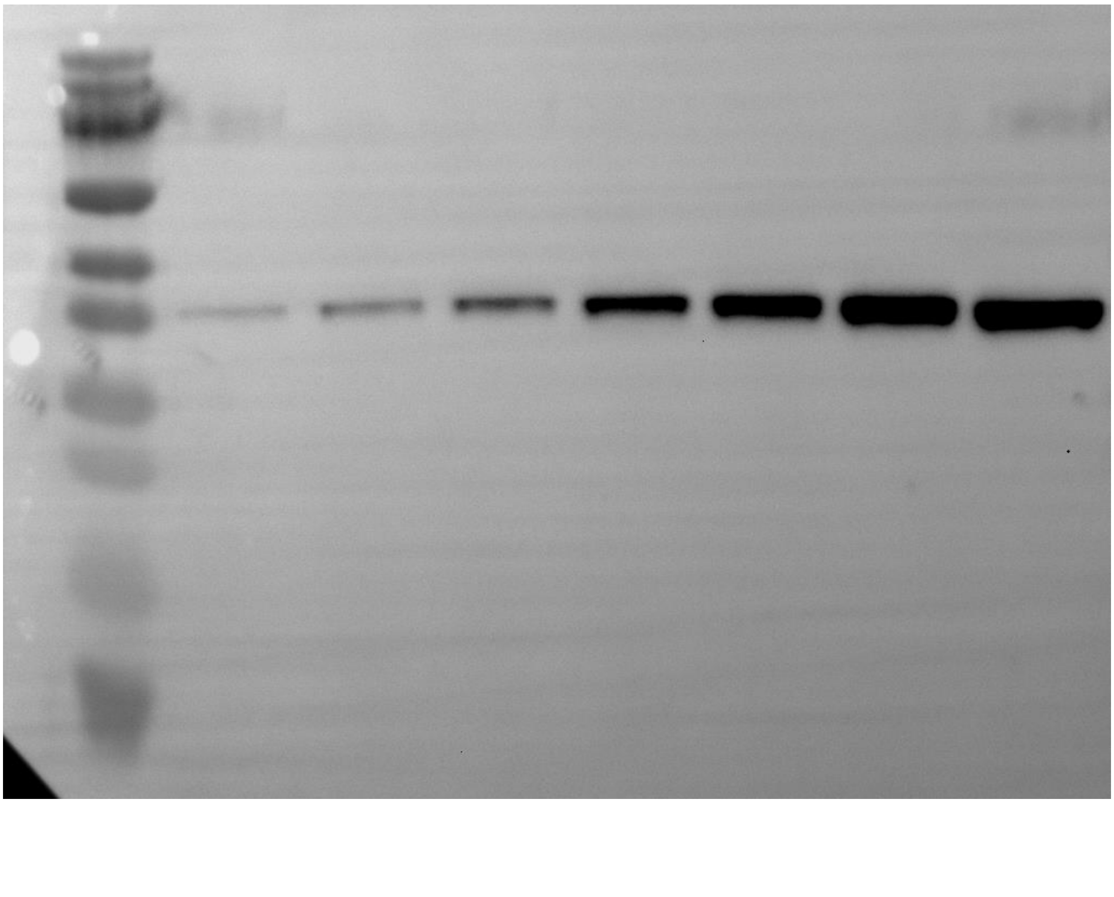

p-IRF3

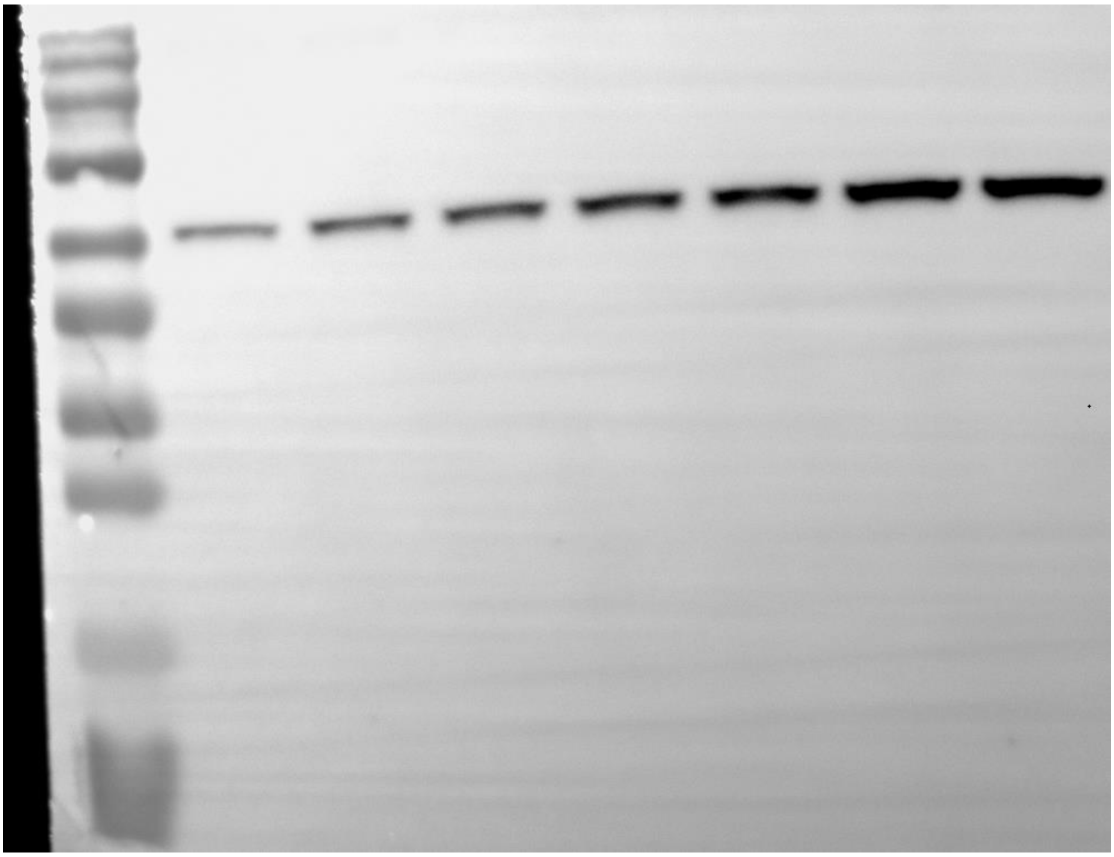

p-TBK1

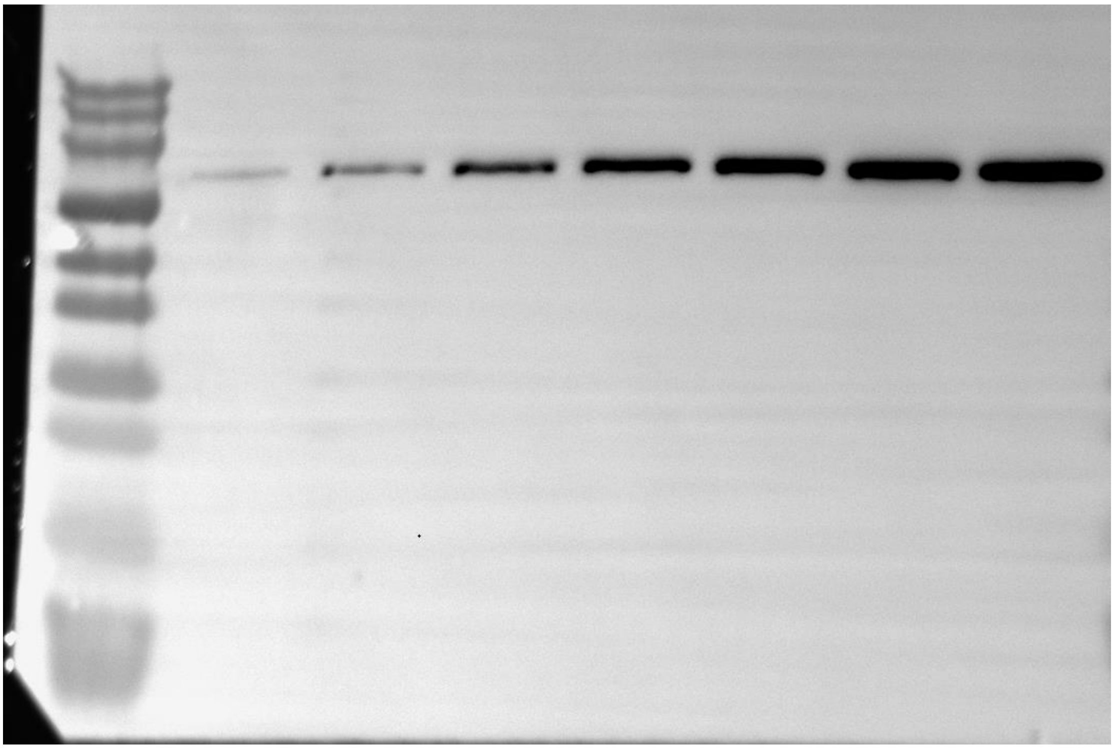

β-actin

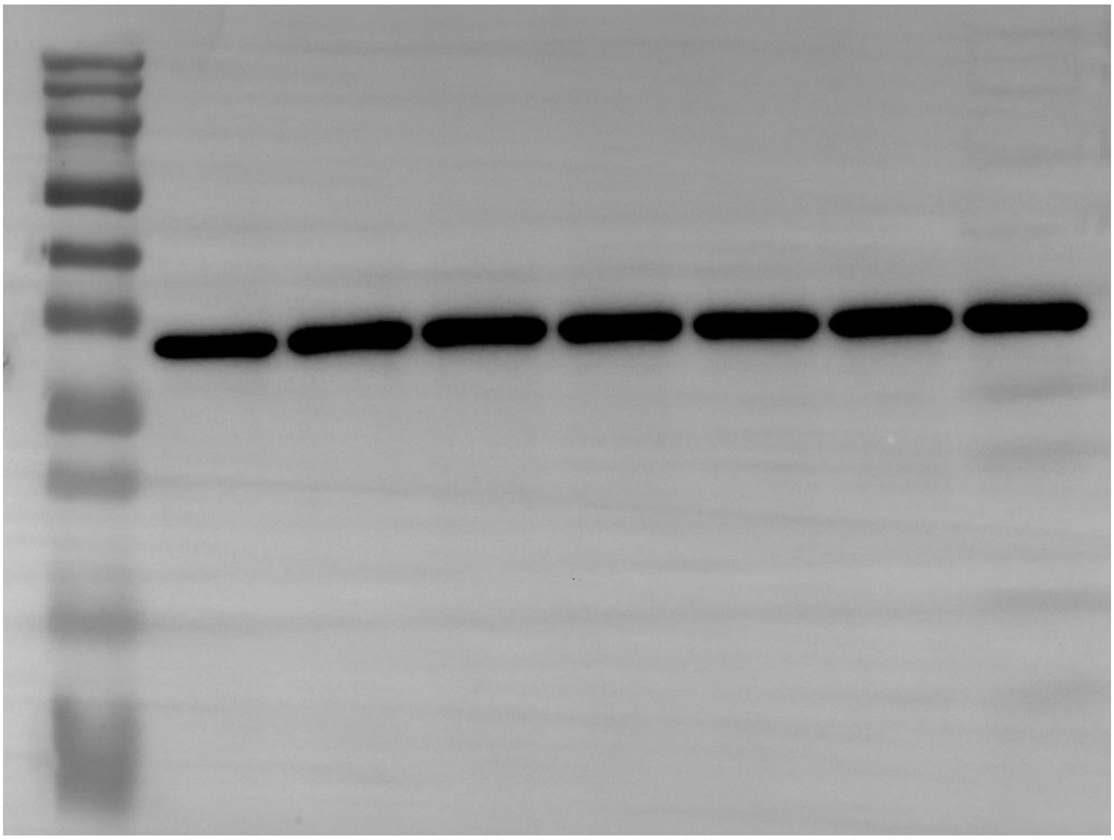

Figure 4A

ATP

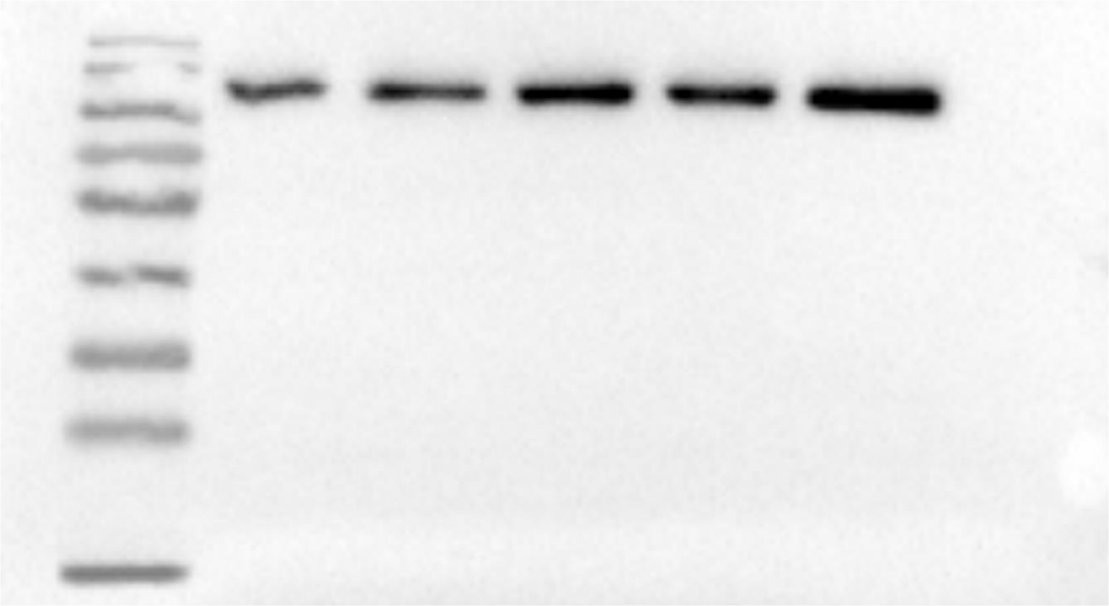

CALR

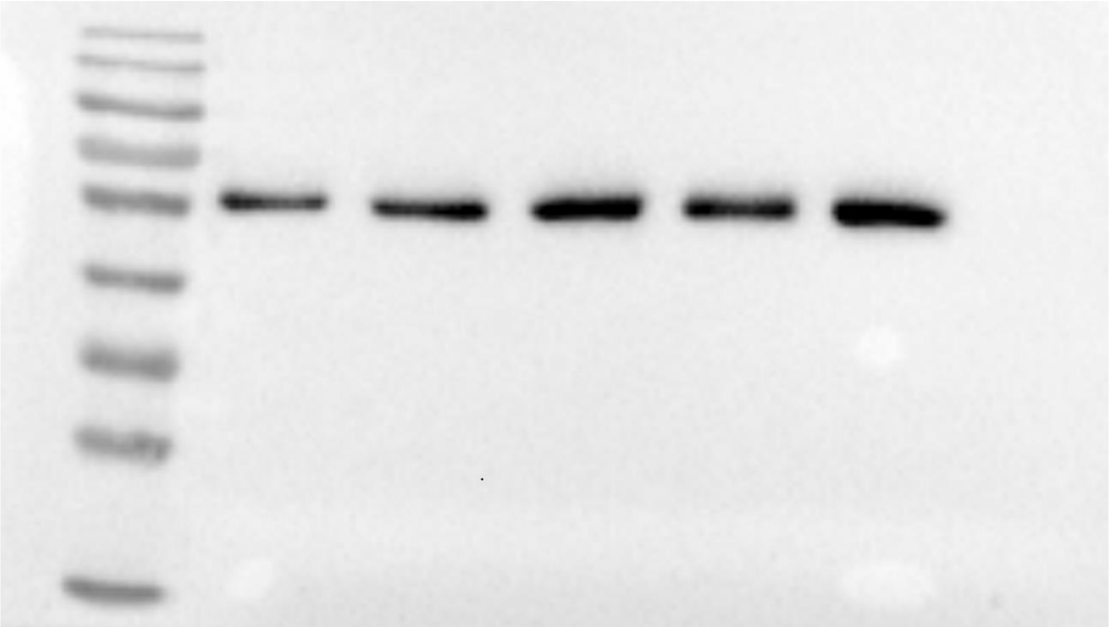

HMGB

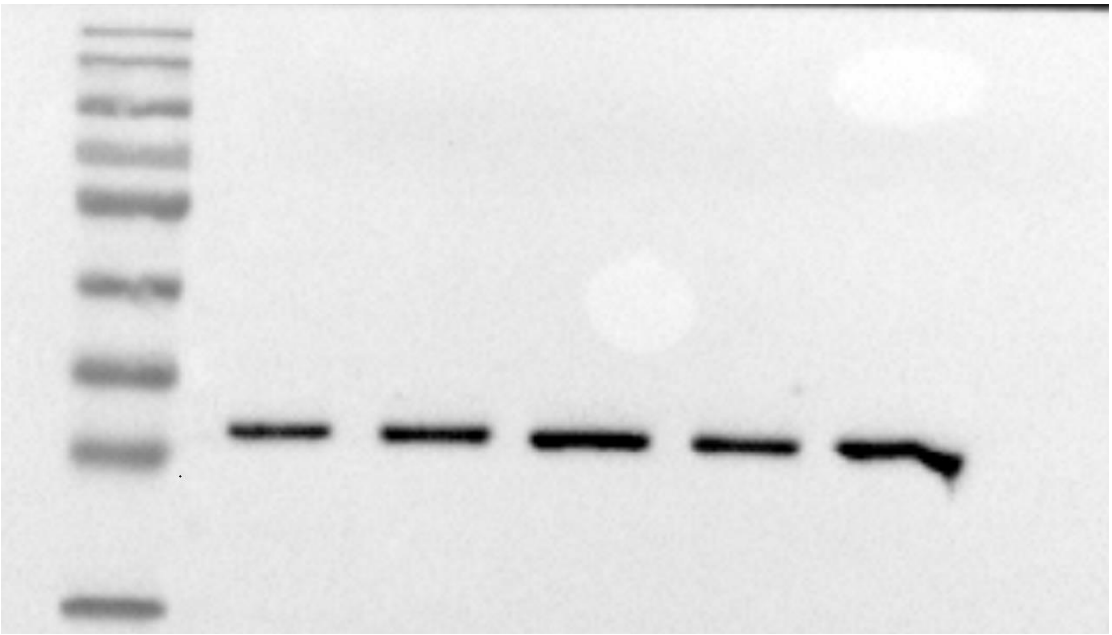

β-actin

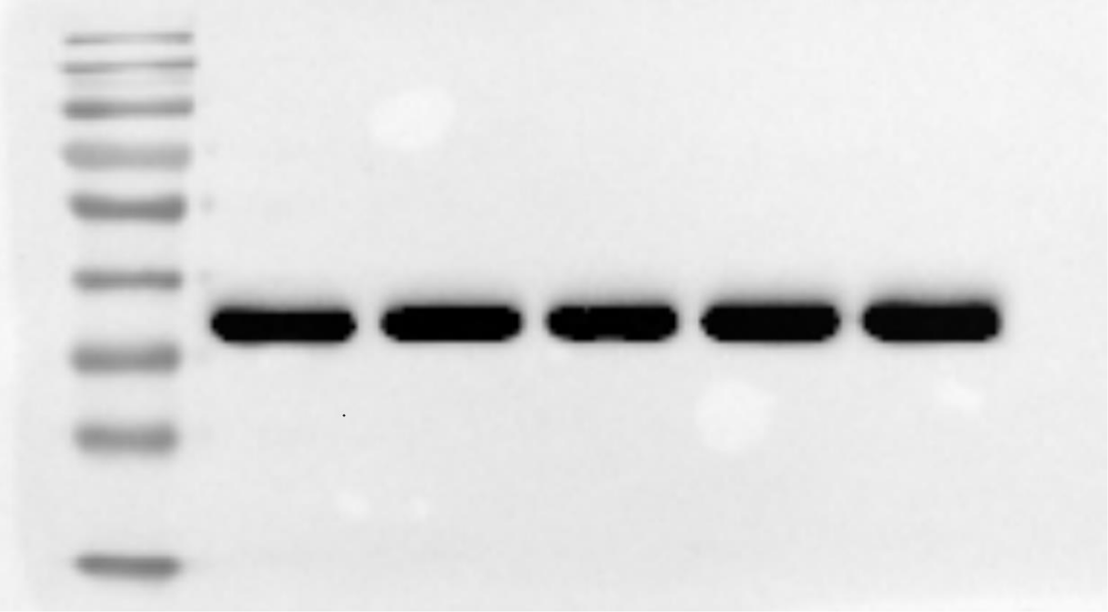

Figure 4C

cGAS

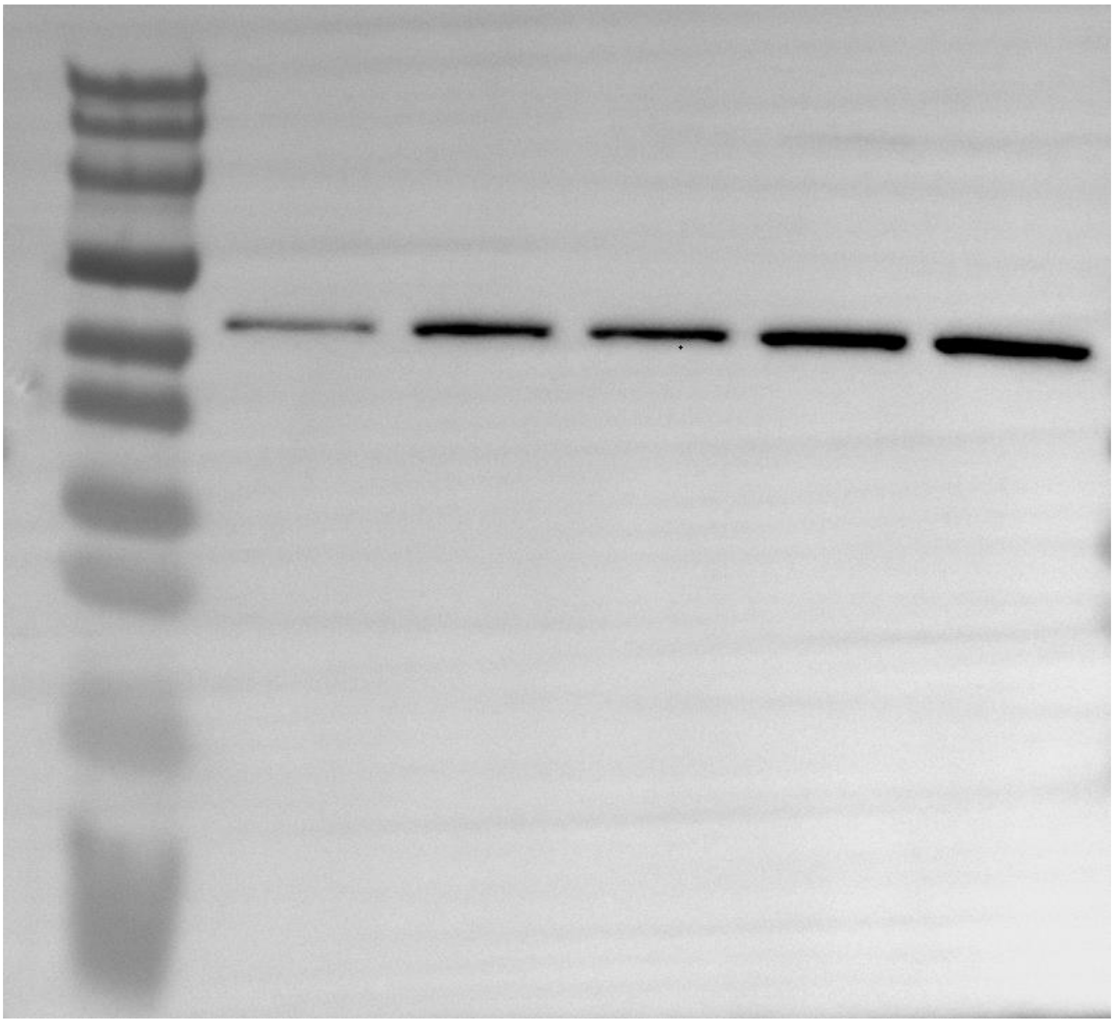

STING

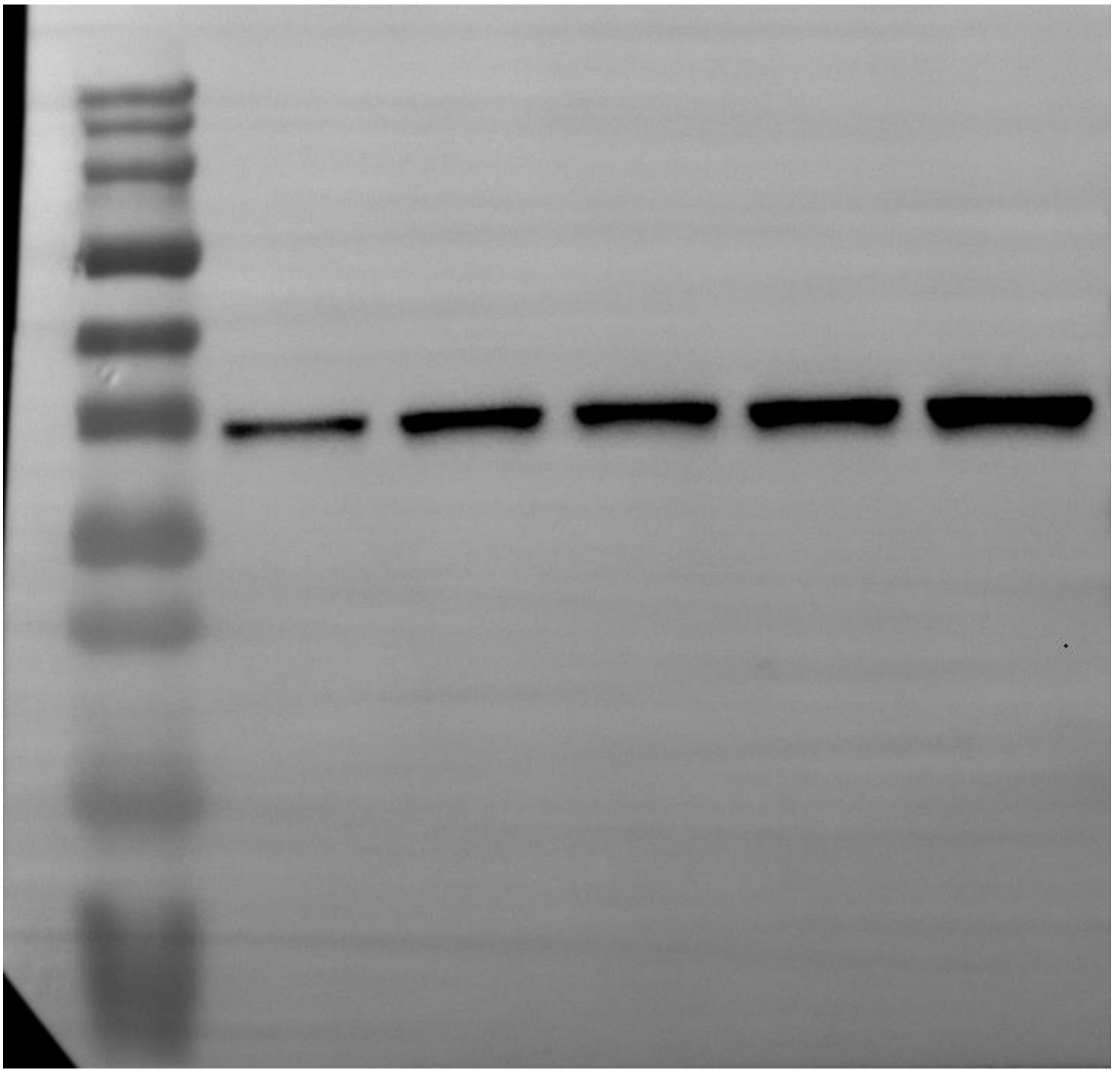

p-IRF3

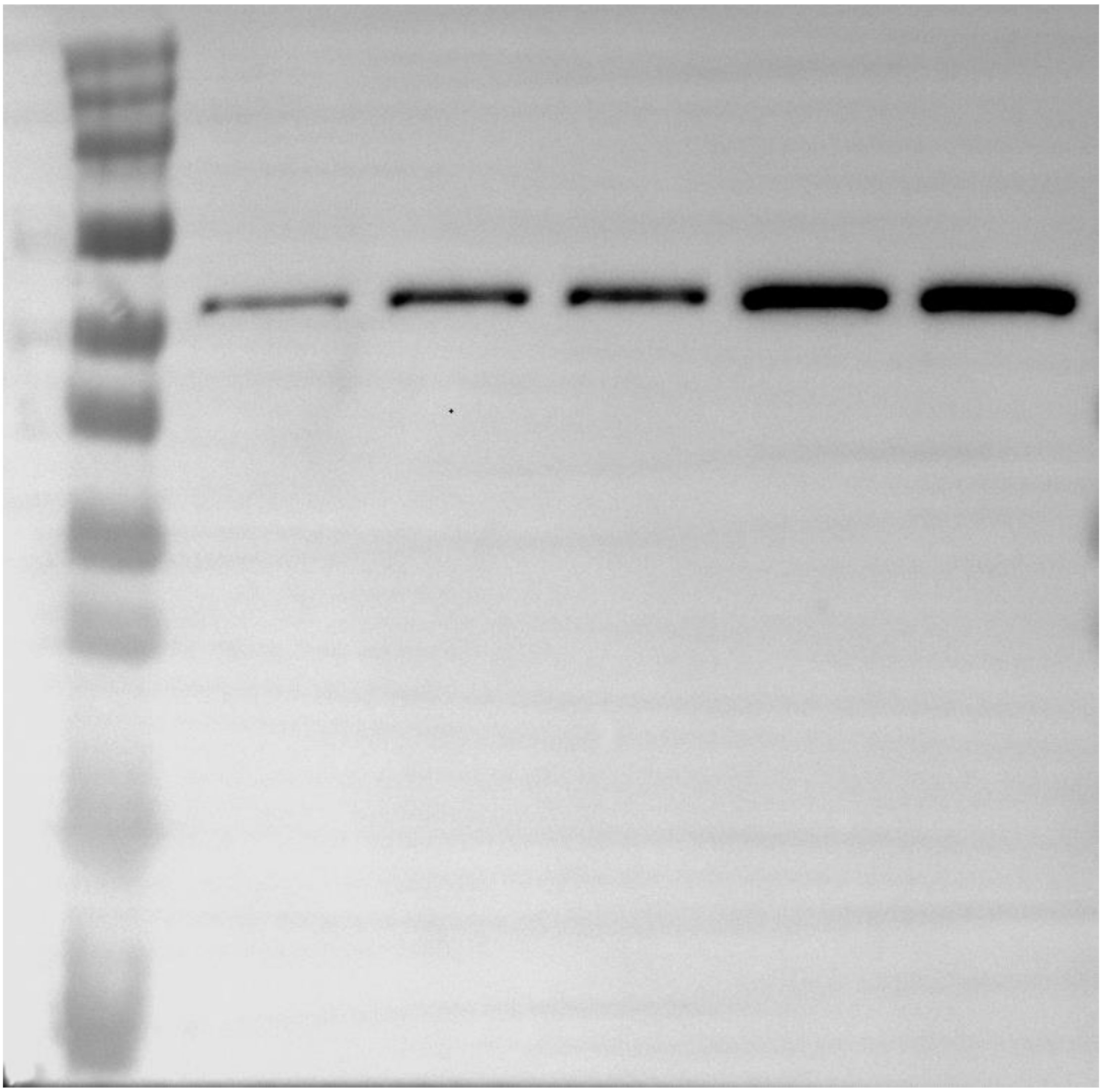

p-TBK1

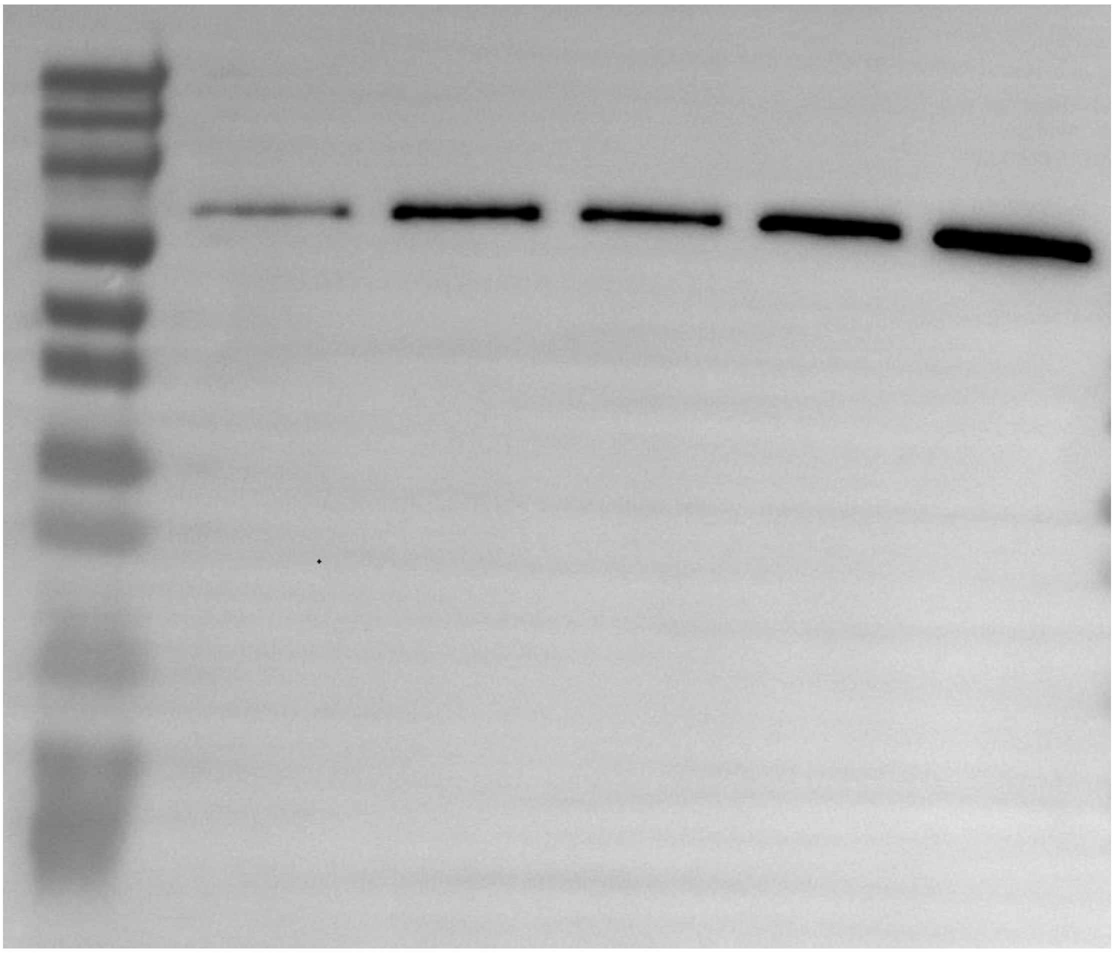

β-actin

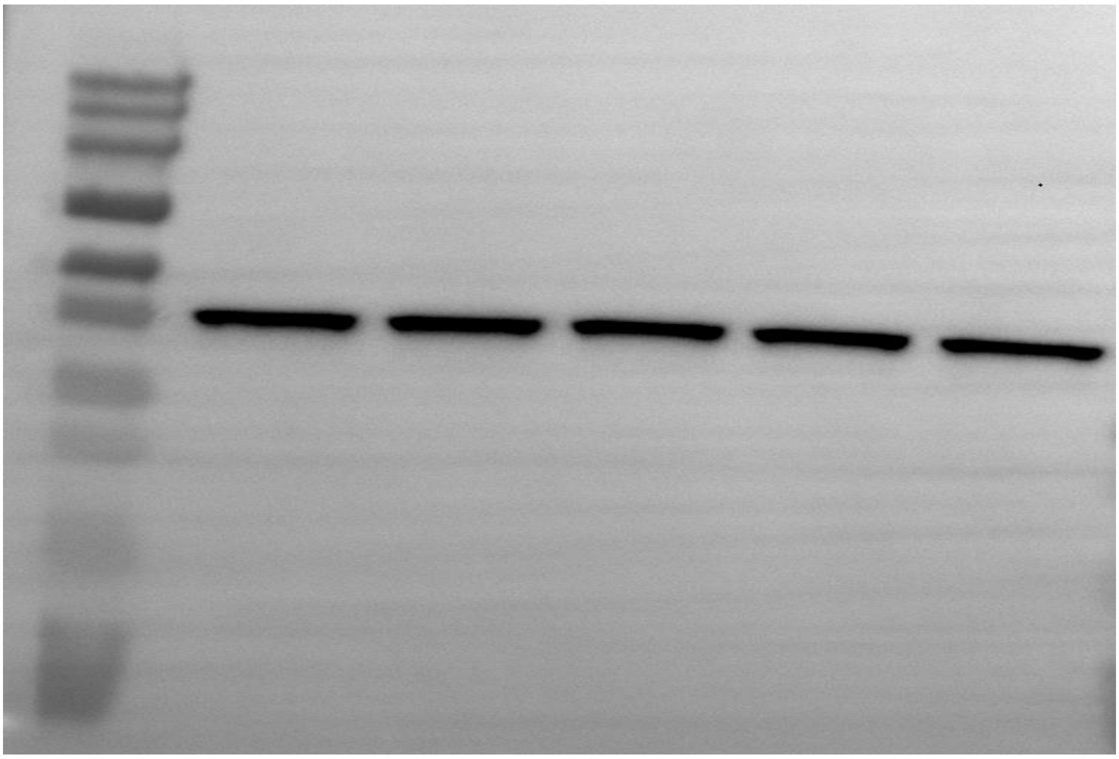

Figure 6B

cGAS

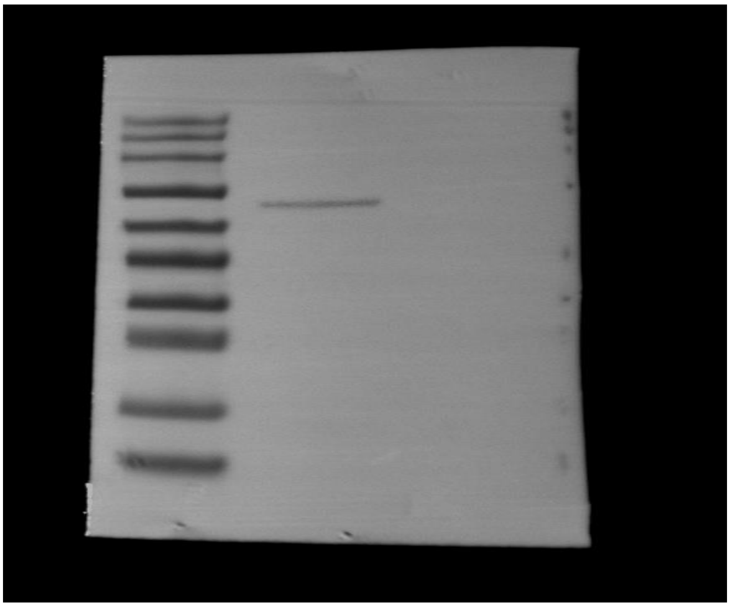

STING

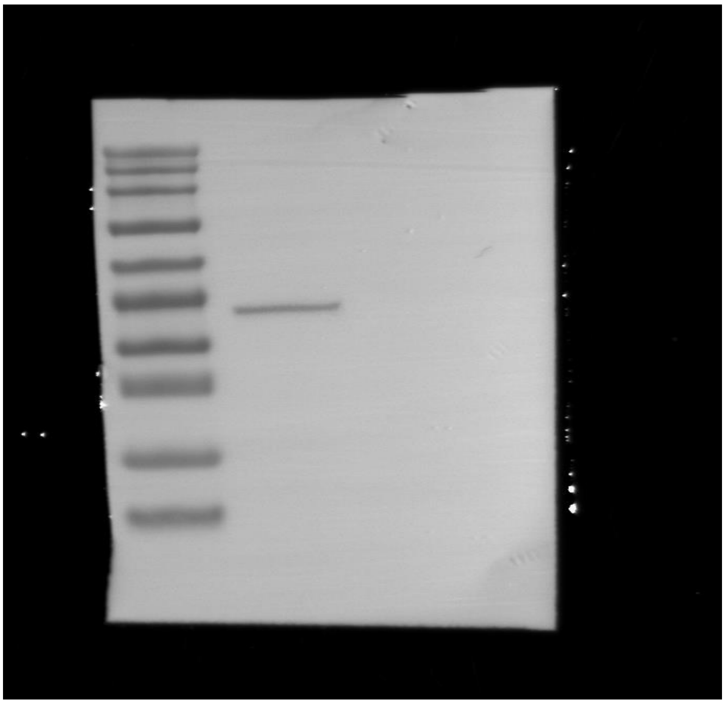

Actin

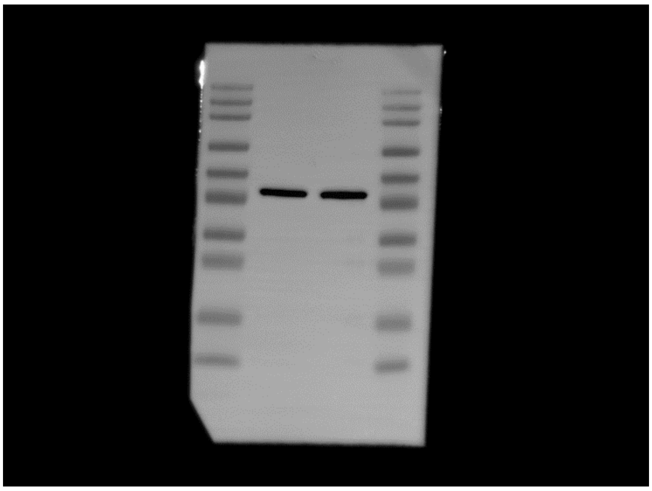

6F

Anneixn a1

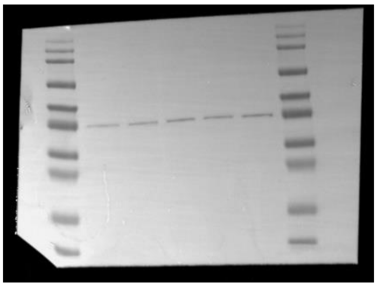

CALR

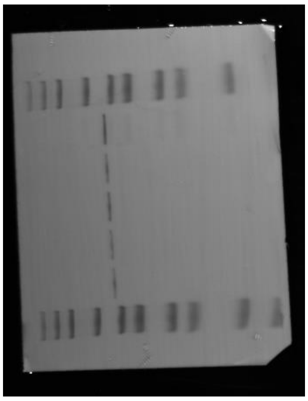

HMGB

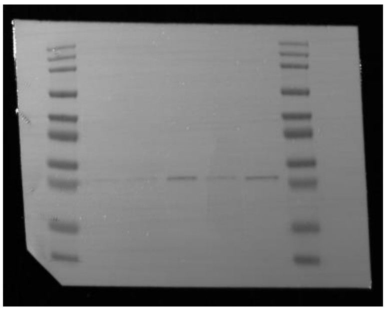

ACTIN

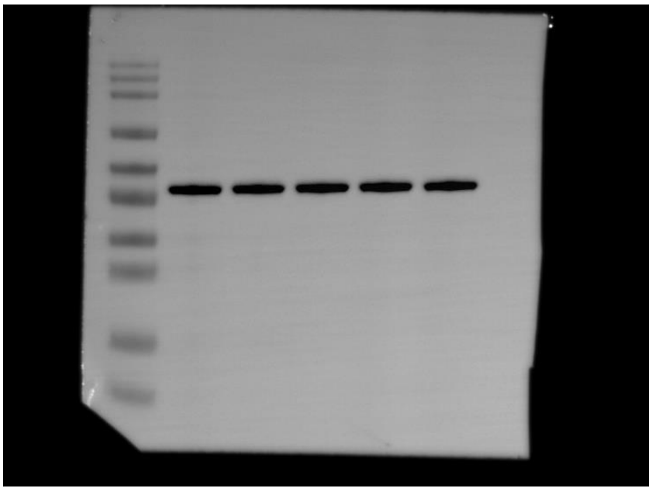

6h

p-TBK1

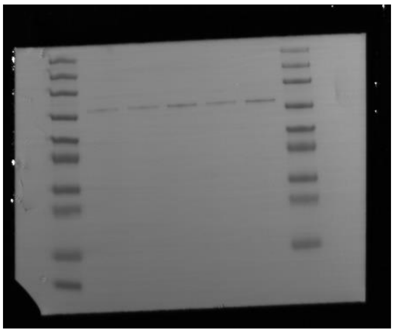

P-IRF3

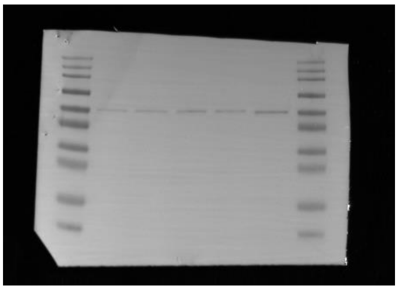

ACTIN

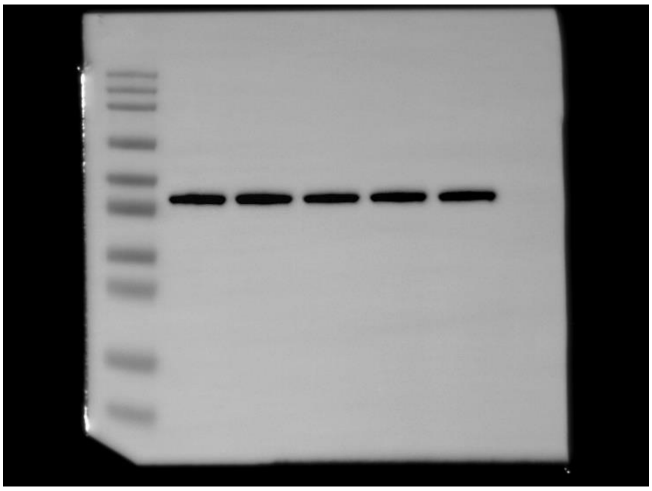

Supplement: Supplementary file 1 — Supplementary file [file 41419_2025_7622_MOESM1_ESM.pdf]
